# Methacrylate-based copolymers as tunable hosts for triplet–triplet annihilation upconversion†

**DOI:** 10.1039/d4ma01221f

**Published:** 2025-01-10

**Authors:** Michael J. Bennison, Abigail R. Collins, Larissa Gomes Franca, Georgina H. Burgoyne Morris, Niamh Willis-Fox, Ronan Daly, Joshua K. G. Karlsson, Bethan L. Charles, Rachel C. Evans

**Affiliations:** a Department of Materials Science and Metallurgy, University of Cambridge CB3 0FS UK rce26@cam.ac.uk; b Institute for Manufacturing, Department of Engineering, University of Cambridge 17 Charles Babbage Rd Cambridge CB3 0FS UK

## Abstract

The ability to convert light to higher energies through triplet–triplet annihilation upconversion (TTA-UC) is attractive for a range of applications including solar energy harvesting, bioimaging and anti-counterfeiting. Practical applications require integration of the TTA-UC chromophores within a suitable host, which leads to a compromise between the high upconversion efficiencies achievable in liquids and the durability of solids. Herein, we present a series of methacrylate copolymers as TTA-UC hosts, in which the glass transition temperature (*T*_g_), and hence upconversion efficiency can be tuned by varying the co-monomer ratios (*n*-hexyl methacrylate (HMA) and 2,2,2-trifluoroethyl methacrylate (TFEMA)). Using the model sensitiser/emitter pair of palladium(ii) octaethylporphyrin (PdOEP) and diphenylanthracene (DPA), the upconversion quantum yield was found to increase with decreasing glass transition temperature, reaching a maximum of 1.6 ± 0.2% in air at room temperature. Kinetic analysis of the upconversion and phosphorescence decays reveal that increased PdOEP aggregation in the glassy polymers leads to a competitive non-radiative relaxation pathway that quenches the triplet state. Notably, the threshold intensity is highly sensitive to the glass transition temperature, ranging from 1250 mW cm^−2^ for PHMA_90_TFEMA_10_ (*T*_g_ = −9.4 °C) to ∼200 mW cm^−2^ for more ‘glassy’ hosts, *e.g.* PHMA_33_TFEMA_67_ (*T*_g_ = 20.1 °C), suggesting the TTA-UC mechanism switches from diffusion-based collisions to triplet exciton migration at localised sensitiser–emitter pairs.

## Abstract 2

Through systematic structural tuning of a family of methacrylate copolymers, we show that triplet–triplet annihilation upconversion of a standard chromophore pair (PdOEP/DPA) can be controlled by the glass transition temperature of the host.

## Graphical abstract

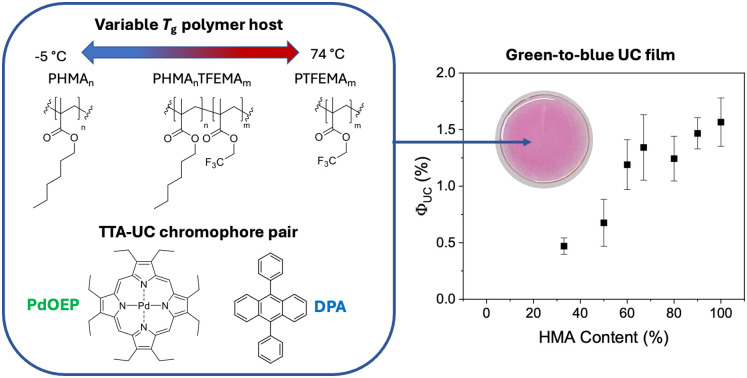

## Introduction

Two low-energy photons can be converted to one high-energy photon using sensitised triplet–triplet annihilation upconversion (TTA-UC).^1,2^ This process has wide-reaching applications including solar energy harvesting,^3–5^ photo-catalysis,^6^ sensors,^7–9^ bioimaging,^10–14^ and anti-counterfeiting.^15^ However, the uptake for some of these applications has been limited by the practicality of incorporating suitable TTA-UC systems in mass-produced devices.^16,17^ The complexity of the TTA-UC mechanism places particular demands on both the luminophores and the host materials in which it is intended to occur.

**Fig. 1:**
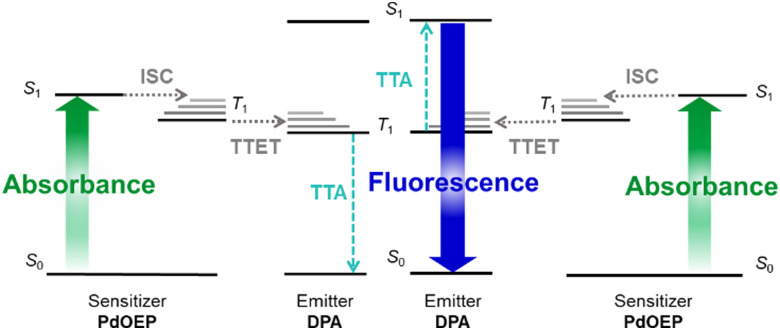
Energy level diagram illustrating the TTA-UC mechanism: absorption of an incident photon (green arrow) by a sensitiser, such as palladium(ii) octaethylporphyrin (PdOEP), resulting in excitation from the ground state (S_0_) to the first singlet excited state (S_1_); intersystem crossing (ISC, grey dashed arrow), to the first triplet excited state (T_1_); triplet–triplet energy transfer (TTET, grey dashed arrow) from the sensitiser to an emitter such as 9,10-diphenylanthracene (DPA); triplet–triplet annihilation (TTA, turquoise dashed arrows) between two T_1_ excited emitters, such that one populates the S_1_ state and the other returns to S_0_; fluorescence (blue arrow) of the S_1_ emitter to return to the ground state.

Fig. 1 illustrates the TTA-UC mechanism, which uses a pair of luminophores: a sensitiser and an emitter. For efficient TTA-UC, the sensitiser should strongly absorb the incident photons to populate the first singlet excited state (S_1_), before undergoing intersystem crossing (ISC) at a high rate to populate the first triplet excited state (T_1_). On collision between a triplet-excited sensitiser and a ground-state emitter, a good energy match between T_1_ states should facilitate effective triplet–triplet energy transfer (TTET). Two triplet-excited emitters may then collide and undergo triplet–triplet annihilation (TTA), such that one emitter populates the higher-energy S_1_ state, while the second relaxes to the ground-state.^18^ Finally, this singlet-excited emitter should fluoresce with a high photoluminescence quantum yield (*Φ*_PL_), emitting a photon at the desired upconverted energy. These multimolecular processes require a high degree of luminophore mobility for the necessary collisions to occur. High rates of chromophore diffusion have been found to be the main factor in enhancement of the TTET efficiency, providing the emitter concentration is not too large (>10^−1^ M).^19^

As a result, efficient TTA-UC has been demonstrated in liquid solutions, with upconversion quantum yields (*Φ*_UC_) exceeding 35% (as a two-photon process *Φ*_UC_ has a maximum cap of 50%).^20,21^ However, potential issues with leakage and solvent evaporation make liquid systems impractical for device integration, so the development of solid-state hosts is desirable. These hosts should facilitate triplet exciton diffusion, allow homogeneous distribution of luminophores without aggregation, and possess high optical transparency, whilst retaining a robust yet flexible mechanical scaffold.^22^ Furthermore, for systems operating in air, ingress of molecular oxygen is detrimental due to quenching of triplet excited states.^23^ Therefore, unless the system is to be fabricated and encapsulated in air-free conditions,^24,25^ an ideal host should also act as an effective barrier to oxygen.^26,27^

Due to their tuneable properties, organic polymers have emerged as promising TTA-UC hosts.^22^ The host performance strongly depends on the glass transition temperature, *T*_g_. Typically the TTA-UC efficiency is maximised using a polymer host whose *T*_g_ is below the required operating temperature.^28^ Chromophore mobility has been shown to have a crucial effect on UC activity, previously shown by lowering the operating temperature of a low *T*_g_ ethyleneoxide-epichlorohydrin polymer host.^28^ Above room temperature, the UC emission was clearly visible, whereas no UC was observed below 280 K which was comparable to the *T*_g_ of the material. It was concluded that for low chromophore concentrations where triplet exciton migration is not possible, fast mobility is essential for successful TTA-UC and the polymer host should be in its rubbery state. Cross-linked elastomers such as polyurethanes and alkyl acrylates are in their rubbery state at usual operating temperatures and provide excellent mechanical stability;^28,29^ however, they are difficult to recycle or reuse, which poses ethical questions around their sustainable use.^30^ Meanwhile, uncrosslinked polymers such as siloxanes operating well above their *T*_g_ behave increasingly as liquids, presenting the same issues of leakage and instability.^31^ To achieve a compromise between stability, efficiency, and processability, the ideal host would therefore be an uncrosslinked polymer whose *T*_g_ is close to, but still below, the intended working temperature.

Methacrylate polymers have been previously investigated as TTA-UC hosts.^32–34^ While upconversion was observed in poly(methyl methacrylate) (PMMA) glasses (a *T*_g_ = 92 °C), a high chromophore concentration (0.005% w/w PdOEP and 25% w/w DPA) was required.^34^ The strategy of increasing emitter concentration can lead to aggregation and subsequent quenching of emission.^33^ Polyacrylate elastomers of decreasing *T*_g_ were investigated by Monguzzi *et al.*^29^ at low chromophore concentrations (0.1 mM PdOEP, 10 mM DPA), where it was concluded that the diffusion length of excited chromophores is extended as the rigidity of host decreases. The host with the lowest *T*_g_ of −62 °C achieved the highest TTA-UC efficiency of 21%, highlighting the importance of the host state on overall performance.

Previous studies have focused on the development of a specific host system whose properties are tuned to suit specific operation conditions.^19,35,36^ Here, we take an alternative approach, in which we design a series of structurally-related polymer hosts, whose properties can be tuned to the device requirements. This is achieved through copolymerisation of a low *T*_g_ methacrylate monomer with a significantly higher *T*_g_ comonomer such that, by varying the ratio, a spectrum of intermediate *T*_g_ values may be targeted. The resultant copolymer hosts were doped with the benchmark TTA-UC sensitiser–emitter pair of palladium(ii) octaethylporphyrin (PdOEP) and 9,10-diphenylanthracene (DPA) to assess the correlation between the thermal properties of the host, and the TTA-UC characteristics. Using a combination of steady-state and time-resolved spectroscopic analysis, we propose that the TTA-UC mechanism transitions from diffusion-based collisions to triplet exciton migration at localised sensitiser as the glass transition temperature of the host increases.

## Results and discussion

### Design of the copolymer host system

**Fig. 2:**
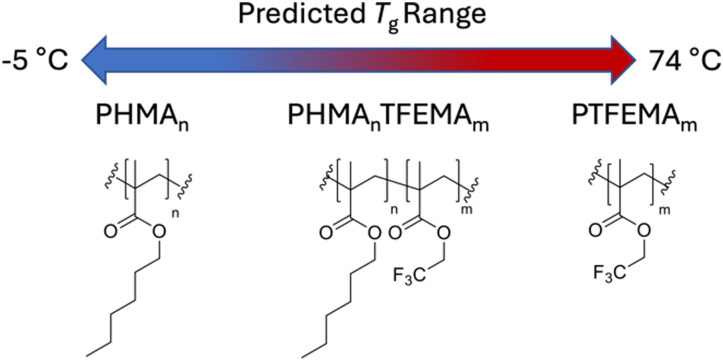
Chemical structures of the homopolymers, PHMA and PTFEMA, and the resultant copolymer poly(*n*-hexyl methacrylate-*co*-2,2,2-trifluoroethyl methacrylate) (PHMA_*n*_TFEMA_*m*_). The monomer ratios, *n* and *m*, were varied to access a range of glass transition temperatures, *T*_g_.

The choice of host system was determined by several requirements: (i) the polymer class should satisfy the outlined general host demands for TTA-UC; (ii) there should be a wide selection of functional monomers available; (iii) the identified monomers should be compatible with copolymerisation. The methacrylate homopolymer family has previously been demonstrated to be a suitable host for TTA-UC, with *Φ*_UC_ from 0.08–3.5%,^37–39^ primarily using alkyl-functional monomers due to their low glass transition temperatures (*T*_g_).^29^ Moreover, there is a wide variety of available methacrylate monomers suited to copolymerisation, which affords tunability in the *T*_g_. We opted to use *n*-hexyl methacrylate (HMA), and 2,2,2-trifluoroethyl methacrylate (TFEMA) as monomers (Fig. 2), since their respective homopolymers show significant difference in their *T*_g_s (−5 °C and 74 °C, respectively).^40^

**Fig. 3:**
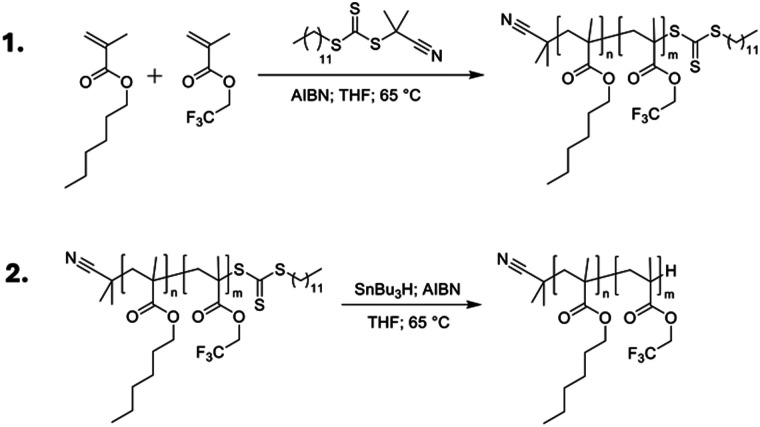
Synthesis of methacrylate copolymers. Step 1: polymerisation of *n*-hexyl methacrylate and 2,2,2-trifluoroethyl methacrylate *via* RAFT polymerisation. Step 2: Radical reduction of trithiocarbonate using AIBN and tributyl tin hydride to remove coloration from the final polymers.

A series of poly(*n*-hexyl methacrylate-*co*-2,2,2-trifluoroethyl methacrylate) copolymers was synthesised using reversible-addition fragmentation transfer (RAFT) polymerisation, using 2-cyanopropan-2-yl dodecyl trithiocarbonate as a chain transfer agent (CTA)^41^ and varying ratios of TFEMA (*m*) and HMA (*n*) to tune the *T*_g_, as outlined in Fig. 3 and Table 1. RAFT polymerisation was chosen to give control over the molecular weight (*M*_w_) of the synthesised polymers, and as such minimise any contribution from *M*_w_ variation to the photophysical properties. All synthesised polymers were in the range 7400–16 400 g mol^−1^ (Table 1). A key drawback of the RAFT method is that the CTA can give strong colouration to the final material, ranging from pink to yellow depending on the exact CTA used.^42^ In our case, the CTA resulted in a strong yellow colouration (see Fig. S4, ESI†), which would lead to parasitic absorption that would be detrimental to the TTA-UC efficiency. To overcome this, the CTA end group was removed post-polymerisation *via* reduction with azobisisobutyronitrile (AIBN) and tributyltin hydride to leave a single hydrogen as the chain end (Fig. 3, step 2). This mechanism has been reported to have a quantitative yield and to be particularly effective for methacrylic polymers.^43^ The resulting end-reduced polymers showed no coloration and retained good optical clarity (Fig. S4, ESI†). The final copolymers are denoted as PHMA_*n*_TFEMA_*m*_, where *n* and *m* are the molar percentages for HMA and TFEMA, respectively. All polymers, before and after end-reduction were fully characterised by ^1^H and ^13^C nuclear magnetic resonance (NMR) spectroscopy (Tables S1, S2 and Fig. S8–S39, ESI†) and size-exclusion chromatography (SEC, Fig. S40, ESI†). Full details of the synthetic methodology and characterisation data are available in the ESI† (Sections 1–3).

**Table 1:** Thermal properties and molecular weights of methacrylate (co)polymers

Polymer	Form	*T*_g(Calc)_ a (°C)	*T*_g_ b (°C)	*M*_n_ c (g mol^−1^)	*M*_w_ d (g mol^−1^)	*Đ* e
PTFEMA_100_	Glassy solid	74	43.3	7400	10 300	1.40
PHMA_33_TFEMA_67_	Glassy solid^f^	43	20.1	10 200	13 600	1.33
PHMA_50_TFEMA_50_	Rubbery solid	29	16.6	10 700	14 300	1.34
PHMA_60_TFEMA_40_	Highly viscous liquid	22	10.4	11 500	15 000	1.31
PHMA_67_TFEMA_33_	Highly viscous liquid	17	1.0	12 100	15 600	1.29
PHMA_80_TFEMA_20_	Viscous liquid	8	−8.49	14 300	18 200	1.27
PHMA_90_TFEMA_10_	Viscous liquid	1	−9.42	15 700	19 500	1.24
PHMA_100_	Viscous liquid	−5	−10.26	16 400	20 500	1.25

a Calculated glass transition temperature (from eqn (1)).

b Measured glass transition temperature (from DSC).

c Number-average molecular weight (from SEC).

d Weight-average molecular weight (from SEC).

e Dispersity, calculated as *M*_w_/*M*_n_.

f Form varied with laboratory temperature due to overlap with *T*_g_.

### Thermal and flow properties

The ratios of HMA and TFEMA used to fabricate the copolymers were identified using the using the Fox equation (eqn (1)),^44^ which predicts the expected *T*_g_ of the copolymer based on the weight fractions *W*_*i*_ of each comonomer and the glass transition temperatures of their respective homopolymers (*T*_g,*i*_):1
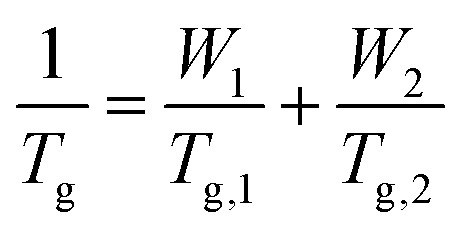
It should be noted that the similar molecular weights of HMA (170.25 g mol^−1^) and TFEMA (168.11 g mol^−1^) mean the weight fractions are approximately equal to the molar fractions of each comonomer, and will therefore be referred to as “fraction” for simplicity. The Fox equation predicts a decrease in *T*_g_ with the increasing fraction of HMA, notably dropping to *T*_g_ ∼ 22 °C with the inclusion of 60% HMA (Table 1), suggesting this is the minimum weight percentage required for a high-diffusion TTA-UC host. Qualitative inspection of the synthesised polymers supports this predicted transition point, with polymers containing 67–100% HMA existing as highly viscous liquids, while <50% resulted in glassy solids. Notably, PHMA_33_TFEMA_67_ exhibited a variable form, from a highly viscous liquid to a glassy solid as the laboratory temperature varied (between 18–24 °C).

Differential scanning calorimetry (DSC) was performed to assess the validity of the predicted to experiment *T*_g_ values. As shown in Table 1, the measured *T*_g_ values are all notably lower than those predicted, which can be rationalised based on the relatively low *M*_w_ of the polymers, which results in a higher free volume due to the chain ends and hence lower *T*_g_ than those calculated for infinite chains. While these lower *T*_g_ values will result in a shift towards more liquid-like behaviour for all copolymers, the compositional region in which the behaviour of the synthesised polymers shifts from glassy to viscous liquid remains around 40–50% HMA. Crucially, the trend is as predicted: the *T*_g_ decreases as HMA content is increased, such that the PHMA_*n*_TFEMA_*m*_ series spans a range of properties from glassy solids to viscous liquids at room temperature.

### Steady-state optical properties

**Fig. 4:**
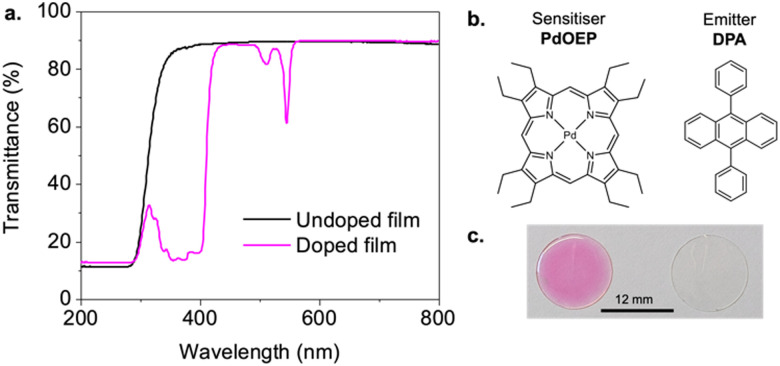
Optical properties of methacrylate copolymer hosts. (a) Transmittance spectra of undoped (black) and doped (pink) PHMA_50_TFEMA_50_ copolymer film. (b) Chemical structures of sensitiser PdOEP and emitter DPA. (c) Photographs of a lumophore-doped film (left), and undoped film (right).

Polymers were doped with PdOEP (0.3 mM) and DPA (30 mM) as the TTA-UC sensitiser–emitter pair and cast as thick films (∼200 μm) on glass substrates. This chromophore pair has been extensively studied for green-blue upconversion, providing a useful benchmark to assess the host performance.^35,45,46^ The sensitiser-to-emitter ratio was selected based on previous studies using samples of similar thickness to facilitate comparison.^26,29^Fig. 4a presents the transmittance spectra for both undoped and doped PHMA_50_TFEMA_50_ copolymer films (see Fig. S42–S44, ESI† for corresponding data for all other samples). The undoped copolymers exhibit good optical transmittance in the visible range (85–95%). When doped with the TTA-UC luminophore pair, the polymer films retain high transmittance above 600 nm, but absorb strongly from 490–560 nm (PdOEP, Q band) and 320–420 nm (DPA S_0_ → S_1_ and PdOEP Soret band) as expected. The individual absorption and emission spectra of DPA and PdOEP in tetrahydrofuran solutions (5 μM) are provided in Fig. S45, ESI† for reference.

**Fig. 5:**
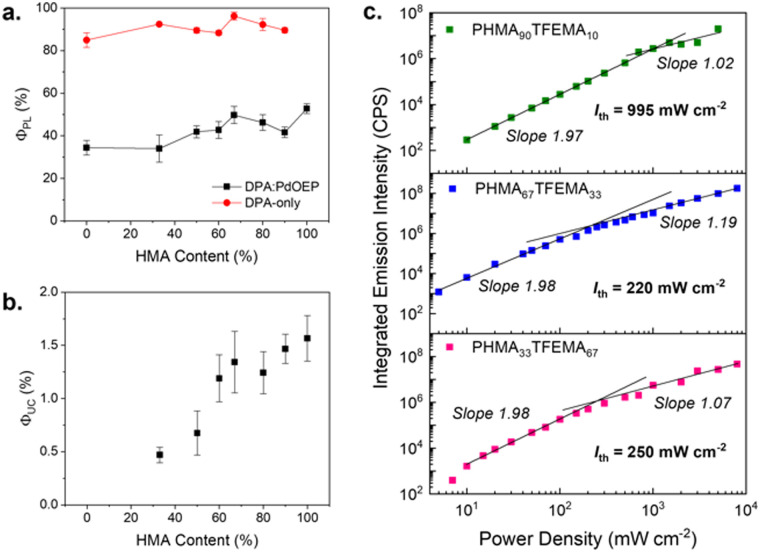
Upconversion and photoluminescence of methacrylate copolymers. (a) Photoluminescence quantum yield of the emitter DPA with sensitiser present (red circles) and without sensitiser (black squares) with increasing hexyl content of the copolymer, *λ*_ex_ = 375 nm, *λ*_em_ = 380–530 nm. (b) Upconversion quantum yield at excitation intensity of 1 W cm^−2^, *λ*_ex_ = 532 nm, *λ*_em_ = 380–500 nm. (c) Threshold intensity was determined from a logarithmic plot of integrated UC emission intensity at varying laser power (532 nm) for PHMA_90_TFEMA_10_ (green), PHMA_67_TFEMA_33_ (blue), and PHMA_33_TFEMA_67_ (pink).

Photoluminescence quantum yields (*Φ*_PL_) were measured for polymer films doped with 30 mM of DPA (DPA-only, Table S4, ESI†) and for the TTA-UC doped films (PdOEP:DPA, Table 2) to understand the impact of the host. In DPA-only samples, upon direct excitation at 375 nm, the *Φ*_PL_ values exceeded 85% for all hosts (Fig. 5a), with the PHMA_67_TFEMA_33_ sample reaching 96%, comparable to that of DPA in solvents.^47^ In contrast, the PdOEP:DPA-doped polymer films exhibited a significant decrease in *Φ*_PL_, ranging from 33 to 50% due to parasitic absorption from the Soret band of PdOEP. As shown in Fig. 5a, the *Φ*_PL_ increases with increasing HMA content, from 49.7% in PHMA_67_TFEMA_33_ to 52.8% in PHMA_100_ - the lowest *T*_g_ matrix.

**Table 2:** Key photophysical properties of PdOEP : DPA (0.3 mM : 30 mM) doped PHMA_*n*_TFEMA_*m*_ hosts

Polymer	*Φ*_PL_ a (%)	*Φ*_UC_ b (%)	UC lifetimes (collection at 440 nm)^c^				Phosphorescence lifetimes (collection at 660 nm)^d^			
			*τ*_1_ (ms)/*f*_1_ (%)	*τ*_2_ (ms)/*f*_2_ (%)	〈*τ*_UC_〉 (ms)	*χ* ^2^	*τ*_1_ (ms)/*f*_1_ (%)	*τ*_2_ (ms)/*f*_2_ (%)	〈*τ*_phos_〉^a^ (ms)	*χ* ^2^
PHMA_100_	53 ± 2	1.6 ± 0.2	1.1/29	3.1/71	2.52	1.085	0.36/22	2.3/78	1.88	1.453
PHMA_90_TFEMA_10_	42 ± 3	1.4 ± 0.1	0.2/38	0.59/62	0.44	1.381	0.51/17	1.4/83	1.24	1.362
PHMA_80_TFEMA_20_	46 ± 4	1.2 ± 0.2	0.18/42	0.48/58	0.35	1.365	0.29/11	1.4/89	1.27	1.413
PHMA_67_TFEMA_33_	50 ± 4	1.3 ± 0.2	0.18/35	0.61/65	0.46	1.522	0.15/22	1.5/78	1.21	1.451
PHMA_60_TFEMA_40_	43 ± 4	1.2 ± 0.2	0.15/41	0.43/59	0.32	1.388	*	*	*	*
PHMA_50_TFEMA_50_	42 ± 3	0.7 ± 0.2	0.16/39	0.45/61	0.34	1.247	*	*	*	*
PHMA_33_TFEMA_67_	34 ± 6	0.5 ± 0.1	0.11/57	0.27/43	0.18	1.358	0.09/29	0.71/71	0.53	1.400
PTFEMA_100_	34 ± 4	—	0.027/72	0.074/28	0.04	1.134	*	*	*	*

a Photoluminescence quantum yield of the emitter DPA (*λ*_ex_ = 375 nm, *λ*_em_ = 380–530 nm).

b Upconversion quantum yield at excitation power intensity of 1 W cm^−2^ (532 nm).

c Lifetime fitting data for UC decay curves (average lifetime (〈*τ*_UC_〉)), individual lifetimes (*τ*_i_) and fractional contributions (*f*_*i*_) and goodness-of-fit (*χ*^2^). *λ*_ex_ = 532 nm, *λ*_em_ = 440 nm.

d Lifetime fitting data for phosphorescence decay curves (average lifetime (*τ*_Phos_)) individual lifetimes (*τ*_i_) and fractional contributions (*f*_*i*_) and goodness-of-fit (*χ*^2^). *λ*_ex_ = 532 nm, *λ*_em_ = 660 nm. *There is no detectable signal.

### Upconversion characterisation

To quantify the upconversion activity in the different polymer hosts, the upconversion quantum yield was measured (see Section 2.6.1, ESI†). The *Φ*_UC_ can be expressed as:^35^

2 *Φ*_UC_ = ½*fΦ*_ISC_*Φ*_TTET_*Φ*_TTA_*Φ*_PL_

where *Φ*_ISC_, *Φ*_TTET_, *Φ*_TTA_ and *Φ*_PL_ are the quantum yields for ISC, TTET, TTA, and emitter photoluminescence, respectively. The factor *f* represents the probability that a singlet excited state is produced per TTA step.^48^ Note that we will use the conventional description for reporting *Φ*_UC_, which is capped at 50% due to the factor of ½ in eqn (2).

Fig. 5b illustrates how *Φ*_UC_ varies as a function of HMA content in the polymer. The increase in *Φ*_UC_ with HMA content can be attributed to the increase in chromophore mobility with decreasing *T*_g_, with the highest *Φ*_UC_ (1.6 ± 0.2%) obtained for PHMA_100_, the most liquid-like host. A transition can also be seen from a steep increase at lower HMA content to a shallower slope above ∼67% HMA. This corresponds to the transition in host properties, from glassy solids with *T*_g_s above room temperature, to viscous liquids with sub-ambient *T*_g_ values. For the more liquid-like hosts, the diffusivity will be higher, and hence less limiting to the upconversion efficiency. Notably, PHMA_67_TFEMA_33_ presents promising results, as it combines a *Φ*_UC_ of 1.3 ± 0.3%, comparable to those in lower-*T*_g_ hosts, with solid-like behaviour. This is preferable for applications at and above room temperature, compared to more liquid films like those of PHMA_100_. While these efficiencies are below that of the highest performing acrylate hosts of 21%,^29^ the purpose of this study is to investigate the tunability of host properties *via* copolymerisation to suit a variety of applications at varying operating temperatures. Such studies in methacrylate polymers are usually conducted in melt-processed glassy matrixes with high chromophore concentrations *via* triplet exciton migration,^32–34^ which rely on close chromophore proximity, and are avoided in the low *T*_g_ host and mechanism presented here.

### Threshold intensity

Fig. 5c shows the threshold intensity (*I*_th_) for three PdOEP:DPA-doped copolymers: PHMA_90_TFEMA_10_, PHMA_67_TFEMA_33_ and PHMA_33_TFEMA_67_. These hosts were selected to compare systems with low, ambient and high *T*_g_ values. *I*_th_, also known as the power density threshold, is the excitation at which the *Φ*_TTA_ is 50% of the maximum value.^48,49^ Experimentally, *I*_th_ is determined by locating the point at which the TTA-UC emission intensity shifts from a quadratic (where the slope of the emission intensity relative to power is 2) to a first-order dependence on incident light intensity (where the slope is 1).^50^ Systems with lower thresholds are generally favoured and will convert more efficiently under low-power sources such as natural sunlight (100 mW cm^−2^).^48,51^ It should be noted that, in our systems, at laser power densities >500 mW cm^−2^, photobleaching occurred before the full emission spectrum could be measured which led to a plateau at high intensities, which is not an uncommon issue with this measurement.^52^ Photodegradation at high power was quantified for each film (Fig. S46, ESI†), where copolymers show a higher resistance to degradation than the mono-polymers. All samples showed fast photodegradation at very high laser power (4 W cm^−2^) as demonstrated by the decreased UC emission intensity and the appearance of clear spot on the film at the point of laser incidence.

For the more liquid-like PHMA_90_TFEMA_10_ (*T*_g_ = −9.4 °C) the transition from quadratic to first-order dependence transition is clearly discernible and corresponds to *I*_th_ = 995 mW cm^−2^. In contrast, for the more ‘glassy’ hosts, PHMA_33_TFEMA_67_ (*T*_g_ = 20.1 °C) and PHMA_67_TFEMA_33_ (*T*_g_ = 1.0 °C), this transition is more subtle and leads to significantly lower *I*_th_ values, around 220–250 mW cm^−2^. This large decrease in the *I*_th_ value indicates that changing the *T*_g_ of the host can completely alter the TTA-UC mechanism. Low threshold intensities have been reported for crystalline TTA-UC materials, which often exhibit large triplet exciton diffusion lengths of the acceptor molecules due to localised chromophore aggregation.^53–56^ It seems plausible that increased chromophore aggregation will occur in the higher *T*_g_ hosts (see following section), which would support a localised exciton diffusion mechanism and the observed decrease in *I*_th_. Note that although the steady-state photoluminescence spectra indicate a reduction in the first vibronic peak due to reabsorption (Fig. S47, ESI†), lifetime measurements suggest DPA aggregation also makes a contribution (Fig. S48 and Table S5, ESI†). Compared to the monoexponential decay of DPA in dilute THF solution, DPA-only doped methacrylate copolymer films show a biexponential decay, where the short lifetime, *τ*_1_ (∼5 ns) corresponds to quenched species due to aggregation, and *τ*_2_ (∼10 ns) exhibits a longer lifetime associated with the reabsorption effect. Importantly, the contribution of *τ*_1_ is significantly higher for DPA-only PTFEMA_100_, supporting the argument that stronger aggregation is observed in the high *T*_g_ hosts. In contrast, in “soft” hosts, such as PHMA_90_TFEMA_10_, Brownian diffusion of the acceptor molecules is expected to determine and limit the maximum achievable triplet exciton diffusion length of the acceptor,^57^ leading to the large *I*_th_. This change in mechanism would be expected to affect multiple parameters, such as the rates of ISC, TTET, and triplet lifetimes.^49^

### Time-resolved emission measurements

**Fig. 6:**
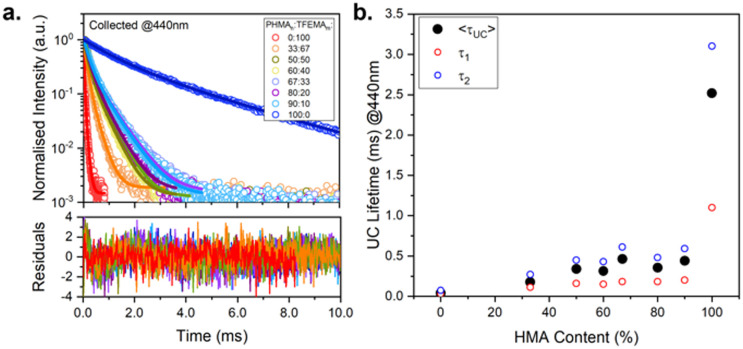
Upconversion kinetic studies on PdOEP : DPA (0.3 mM : 30 mM) doped in different PHMA_*n*_TFEMA_*m*_ host matrices. (a) Upconversion decay traces (open symbols) and biexponential decay fits (solid lines) and corresponding residuals. *λ*_ex_ = 532 nm, *λ*_em_ = 440 nm. (b) Corresponding upconversion average lifetimes (filled black circles) and individual lifetime components (*τ*_1_ and *τ*_2_) obtained from decay fits.

Lifetime measurements were performed to gain better understanding of the variation in upconversion behaviour across the series of polymers. The polymers vary significantly in *T*_g_ and, consequently, in viscosity of the host matrix, which is expected to affect the kinetics of excited state decay of both luminophores (DPA and PdOEP). Fig. 6a shows the upconversion decay traces for the series of PdOEP:DPA-doped polymer films (collected at 440 nm). The UC lifetimes (*τ*_UC_) values were estimated by tail-fitting a biexponential decay function. Fig. 6b and Table 2 summarise the average UC lifetimes (〈*τ*_UC_〉), which ranged from 0.04 ms in PTFEMA_100_ to 2.51 ms in PHMA_100_. In agreement with the *Φ*_UC_ values, a significant increase in the 〈*τ*_UC_〉 is observed as the HMA content of the polymer films increased. This suggests that for the viscous liquid film (PHMA_100_), the higher *Φ*_UC_ value is accompanied by a longer 〈*τ*_UC_〉 indicating reduced competitive quenching mechanisms in this matrix.

The average phosphorescence lifetimes in air (〈*τ*_phos_〉, collection at 660 nm, Fig. S48, ESI†) were obtained for the same samples from biexponential tail fits, comprising a short lifetime component, *τ*_1_ ∼0.1–0.5 ms, and a longer-lived component, *τ*_2_ ∼1.4 ms, as summarised in Table 2. We assign *τ*_2_ to the natural phosphorescence lifetime, since in previous studies of PdOEP-only in polymer hosts, a characteristic *τ*_phos_ of 1.4 ms was observed in the absence of oxygen.^58^ The shorter lifetime observed in these PdOEP:DPA-doped polymer samples, *τ*_1_, is assigned to the quenched triplet state, which arises from competing relation pathways such as aggregation-caused quenching, oxygen quenching and TTET. We observed that polymer hosts with particularly low *T*_g_, *e.g.* PHMA_90_TFEMA_10_, exhibit a high fractional contribution of *τ*_2_, even in air. Meanwhile, those with higher *T*_g_ – *e.g.* PHMA_33_TFEMA_67_-show greater fractional contribution from *τ*_1_, leading to a significantly shorter 〈*τ*_phos_〉. This same trend is also observed in the analogous PdOEP-only doped films (Fig. S49 and Table S5, ESI†).

**Fig. 7:**
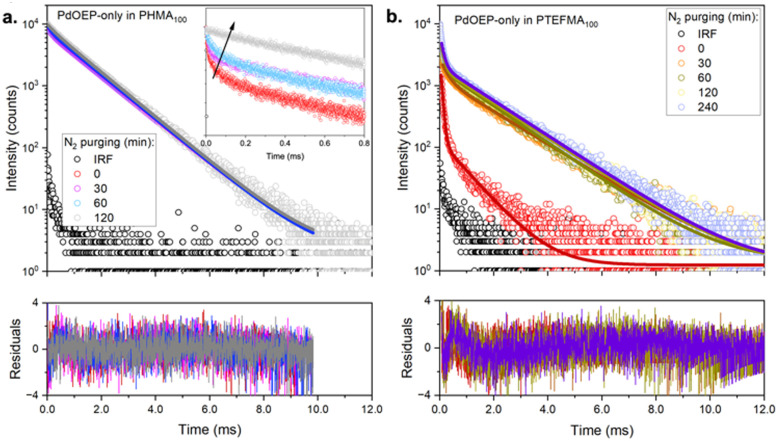
Phosphorescence decay traces of PdOEP-only (0.3 mM) doped in: (a) PHMA_100_ and (b) PTFEMA_100_ under varying durations of nitrogen (N_2_) purging. Inset: Zoom in on the first millisecond regimes to highlight the increase in the shorter lifetime after increased N_2_ purging. Measurements were performed using a 532 nm excitation source and collected at 660 nm.

To further understand the triplet relaxation mechanisms, phosphorescence decay curves (Fig. 7 and Table S6, ESI†) were measured for representative low (PHMA_100_) and high (PTFEMA_100_) *T*_g_ homopolymer hosts doped with PdOEP-only, in air and N_2_ atmospheres. In both cases, *τ*_2_ became more prevalent with deaeration, further supporting the assignment of this component to the natural phosphorescence lifetime of the unquenched species (around 1.2–1.5 ms). Its presence even under ambient conditions suggests that the second coverslip used to aid sample handling prevented further oxygen ingress during the measurement, with any local oxygen already present inside the matrix being rapidly photoconsumed, especially in PHMA_100_ where oxygen is expected to diffuse more readily. On the other hand, for PTFEMA_100_, even in the absence of oxygen, the decay trace still exhibited a significant contribution from *τ*_1_, suggesting that aggregation-caused quenching is a significant relaxation pathway. We note that as DPA is not present in these samples, the contribution from TTET is also excluded. The phosphorescence lifetime analysis thus supports the presence of increased PdOEP aggregation in the high *T*_g_ polymer hosts. Aggregation is also evident in the DPA fluorescence lifetimes (Fig. S48 and Table S5, ESI†), where PdOEP:DPA-doped films exhibit shorter average lifetimes than DPA-only doped films, suggesting additional non-radiative pathways for these samples. This can result in longer triplet exciton diffusion lengths for the acceptors, consistent with the lower threshold intensity observed in these samples.

## Conclusions

We have demonstrated the formation of tunable TTA-UC host materials through RAFT copolymerisation of two structurally-similar methacrylate monomers, HMA and TFEMA. Through variation of the co-monomer ratios, a series of PHMA_*n*_TFEMA_*m*_ copolymers were synthesised, whose glass transition temperatures spanned a range from below to above room temperature. For the model TTA-UC pair (PdOEP:DPA), the upconversion quantum yields increased with decreasing glass transition temperature, across the range 0.5–1.6% in air at room temperature. This is accompanied by a five-fold decrease in the threshold intensity, which we propose is due to increased luminophore aggregation in the higher *T*_g_ hosts, leading to a switch in mechanism from diffusion-based collisions to triplet exciton migration at localised sensitiser–emitter pairs. Notably, this work demonstrates the versatility of copolymerisation to optimise the physical form to meet a required operating temperature or application, without dramatically changing the upconversion efficiency. Further tuning of the chromophore concentrations and limiting of oxygen diffusion and subsequent quenching could be employed in future work to improve efficiency of these hosts. The insight gained through this study will inform the design of next generation TTA-UC hosts, with the aim of increasing the upconversion efficiency in real-world conditions.

## Data availability

Data for this article are available at the University of Cambridge Apollo repository at https://www.repository.cam.ac.uk/home.

## Author contributions

M. J. B.: conceptualisation, methodology, investigation, data curation, formal analysis, writing – original draft, writing – reviewing & editing. A. R. C.: investigation, data curation, formal analysis, writing – original draft, reviewing & editing. L. G. F.: investigation, data curation, formal analysis, writing – original draft, writing – reviewing & editing. G. H. B. M.: data curation, formal analysis, writing – reviewing & editing. N. W. F.: investigation, data curation. R. D.: resources. J. J. G. K.: investigation and data curation. B. L. C.: investigation and data curation. R. C. E.: conceptualisation, project administration, resources, funding acquisition, supervision, writing – reviewing & editing.

## Conflicts of interest

There are no conflicts to declare.

## Supplementary Material

MA-006-D4MA01221F-s001
