# Supplementary material for: Methacrylate-based copolymers as tunable hosts for triplet–triplet annihilation upconversion
Source: Mater Adv. 2025 Jan 10;6(3):1089–96. doi: 10.1039/d4ma01221f (PMC11718357; doi:10.1039/d4ma01221f)
Supplement: MA-006-D4MA01221F-s001 [file MA-006-D4MA01221F-s001.pdf]

Supplementary Information (SI) for Materials Advances.
This journal is © The Royal Society of Chemistry 2024

Supporting Information

Methacrylate-based copolymers as tunable hosts for triplet-triplet annihilation upconversion

Michael J. Bennison,[a] Abigail R. Collins,[a] Larissa Gomes Franca,[a] Georgina H. Burgoyne Morris,[a] Niamh Willis-Fox,[b] Ronan Daly,[b] Joshua K. G. Karlsson,[a] Bethan L. Charles[a] and Rachel C. Evans[a]*

[a] Department of Materials Science & Metallurgy, University of Cambridge, 27 Charles Babbage Road, CB3 0FS, U.K.

[b] Institute for Manufacturing, Department of Engineering, University of Cambridge, 17 Charles Babbage Rd, Cambridge CB3 0FS, UK.

* Corresponding Author: rce26@cam.ac.uk

TABLE OF CONTENTS

1 EXPERIMENTAL METHODS 3

1.1 MATERIALS 3
1.2 SYNTHESIS 3
1.3 PREPARATION OF POLYMER FILMS 8

2 INSTRUMENTATION 9

2.1 NUCLEAR MAGNETIC RESONANCE (NMR) SPECTROSCOPY 9
2.2 SIZE-EXCLUSION CHROMATOGRAPHY (SEC) 9
2.3 DIFFERENTIAL SCANNING CALORIMETRY (DSC) 9
2.4 UV/VIS TRANSMITTANCE AND ABSORPTION SPECTROSCOPY 9
2.5 STEADY-STATE PHOTOLUMINESCENCE (PL) SPECTROSCOPY 10
2.6 UPCONVERSION, PHOSPHORESCENCE AND TIME-RESOLVED EMISSION MEASUREMENTS 10

3 SUPPORTING EXPERIMENTAL DATA 13

3.1 NUCLEAR MAGNETIC RESONANCE SPECTRA 13
3.2 POLYMER SYNTHESIS 14
3.4 MOLECULAR WEIGHT DETERMINATION 30
3.5 THERMAL ANALYSIS 32
3.6 STEADY-STATE OPTICAL PROPERTIES 33
3.7 PHOSPHORESCENCE LIFETIMES 38

4 REFERENCES 40

1 Experimental Methods

1.1 Materials

1-Dodecanethiol (97%), 2,2,2-trifluoroethyl methacrylate (98%), n-hexyl methacrylate (98%) and tri-n-butyltin hydride (97%) were purchased from Alfa Aesar. Carbon disulfide (99.9%), iodine (99.5%), and anhydrous diethyl ether (99.5%) were purchased from Acros Organics. Potassium hydroxide (AR Grade) was purchased from Fisher Scientific. Sodium hydride (60% in mineral oil), 2,2'-azobis(2-methylpropionitrile) (98%), ethyl acetate (AR Grade), dichloromethane (DCM, analytical grade), tetrahydrofuran (THF, ≥99.9%, unstabilised), silver carbonate on celite (50 wt% loading), and 2,3,7,8,12,13,17,18-octaethyl-21*H*,23*H*-porphine palladium (II) (PdOEP, 85%) were purchased from Sigma Aldrich. 9,10-diphenylanthracene (DPA, 99%) was purchased from Alfa Aesar.

2,2,2-Trifluoroethyl methacrylate (TFEMA) and n-hexyl methacrylate (HMA) as purchased were stabilised with 4-methoxyphenol (30-50 ppm and 100 ppm respectively) and the stabiliser removed by elution through a basic alumina column. Tetrahydrofuran was dried over three consecutive batches of activated 3Å sieves. All other chemicals were used as received.

1.2 Synthesis

1.2.1 Chain transfer agent

The chain transfer agent, 2-cyanopropan-2yl dodecyl trithiocarbonate was made in a two-step synthesis according to the method reported by Abel and McCormick.[1]

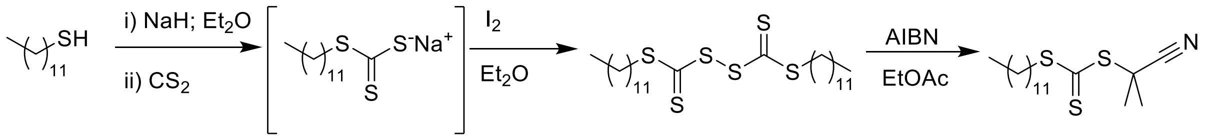

Figure S1. Synthetic route to chain transfer agent.

1.2.2 Bis(dodecyl trithiocarbonate)

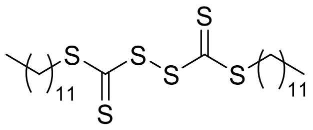

Synthesis based on reported literature procedure.[1] To an oven-dried two-neck round-bottomed flask 1-dodecanethiol (5.00 g; 24.70 mmol) was added and the atmosphere exchanged for nitrogen before the addition of anhydrous diethyl ether (150 mL). Once the thiol was fully

dissolved, sodium hydride (60 wt%; 1.19 g; 29.64 mmol) was added slowly and the reaction cooled to approx. 5 °C before being allowed to stir for 45 minutes. The reaction was cooled to 0 °C before the careful addition of carbon disulfide (1.78 mL; 29.64 mmol), which turned the reaction mixture a vivid yellow, before the reaction was allowed to stir for 1 hour at 0 °C. The resulting thick yellow precipitate was diluted with additional anhydrous diethyl ether (approx. 50 mL) before being isolated *via* filtration. The isolated solid (sodium dodecyl trithiocarbonate) was immediately resuspended in fresh anhydrous diethyl ether (approx. 150 mL) in an oven-dried, nitrogen-flushed two neck round-bottomed flask. To this suspension iodine (3.45 g; 13.59 mmol) was carefully added and the reaction was allowed to stir at room temperature for 18 hours, after which time the reaction had turned a deep orange with a significant quantity of white precipitate. The precipitate was removed *via* filtration, and the filtered solution was washed repeatedly with saturated aqueous sodium thiosulfate (3 x 100 mL). The organic layer was isolated, dried over anhydrous magnesium sulfate, filtered, and the solvent removed under reduced pressure to yield a yellow oil that solidified on standing. Characterisation in accordance with literature report.[1]

^{1}H NMR (400 MHz, $CDCl_3$): δ 3.30 (t, 4H), 1.69 (m, 4H), 1.30 (b, 36H), 0.88 (t, 6H).

1.2.3 2-Cyanopropan-2-yl dodecyl trithiocarbonate

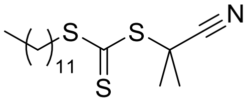

Synthesis based on reported literature procedure.[1] To a dried round-bottomed flask was added bis(dodecyl trithiocarbonate) (4.89 g; 8.81 mmol), this was dissolved in ethyl acetate (200 mL) and the solution deoxygenated by bubbling with nitrogen for approx. 30 minutes before the addition of 2,2'-azobis(2-methylpropionitrile) (2.17 g; 13.22 mmol). The reaction was fitted with a condenser and heated to reflux for 18 hours before being cooled to room temperature and subsequently washed with deionised water (3 x 150 mL) and saturated brine (150 mL). The organic layer was isolated, dried over anhydrous magnesium sulfate and filtered, before removal of the solvent under reduced pressure to yield a yellow oil. Crude product was purified *via* column chromatography (95:5 hexane:ethyl acetate, Rf = 0.35) to yield a yellow oil that solidified on standing (2.19 g; 6.34 mmol; 72%). Characterisation in accordance with literature report.[1]

^{1}H NMR (60 MHz, $CDCl_3$): δ 3.34 (t, 2H), 1.87 (s, 6H), 1.69 (m, 3H), 1.26 (br, 20H), 0.88 (t, 3H)

^{13}C NMR (126 MHz, $CDCl_3$) δ 228.64, 224.97, 39.88, 37.93, 37.01, 32.06, 29.79, 29.78, 29.77, 29.77, 29.73, 29.71, 29.70, 29.63, 29.59, 29.50, 29.49, 29.49, 29.37, 29.32, 29.28, 29.27, 29.21, 29.07, 28.64, 28.16, 27.66, 22.84, 14.27.

1.2.4 RAFT copolymerisation of n-hexyl methacrylate and 2,2,2-trifluoroethyl methacrylate to form poly(*n*-hexyl methacrylate-*co*-2,2,2-trifluoroethyl methacrylate) ($PHMA_nTFEMA_m$)

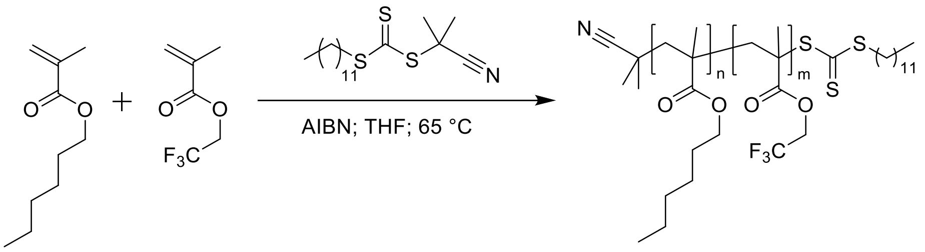

Figure S2. Schematic route to $PHMA_nTFEMA_m$ copolymers.

An oven-dried 50 mL Young's tap ampoule was attached to a Schlenk line and the atmosphere exchanged for nitrogen *via* repeated evacuation/refill cycles before the addition of *n*-hexyl methacrylate (2.32 mL; 11.77 mmol), 2,2,2-trifluoroethyl methacrylate (0.84 mL; 5.88 mmol), 2-cyanopropan-2-yl dodecyl trithiocarbonate (61 mg; 176.5 μmol), anhydrous tetrahydrofuran (17.65 mL), and 2,2'-azobis(2-methylpropionitrile) (5.8 mg; 35.3 μmol). The resulting solution was deoxygenated *via* repeated freeze-pump-thaw cycles, before the solution was sealed under vacuum and heated to 65 °C. The reaction was monitored *via* ^1H NMR spectroscopy and stopped once monomer conversion plateaued (typically 90%+ conversion, see **Table S1**), at which point solution was rapidly cooled to room temperature and opened to air to halt polymerisation. Solvent and unreacted monomer were subsequently removed under reduced pressure to yield a bright yellow polymer. For all polymerisations, an initial monomer:CTA:initiator ratio of 100:1:5 was used, with an initial monomer concentration of 1.0 mol.L^{-1}. The desired copolymer composition was obtained by varying the fraction of HMA and TFEMA in the initial reaction mixture, as shown in **Table S1.** The conversion (%) was obtained by measuring the ratio of integrals for key monomer and polymer peaks in the ^1H NMR spectra. For PHMA the α-CH_2 was tracked, shifting from 4.12 to 3.91 ppm on polymerisation. For TFEMA the trifluoroethyl CH_2 was monitored, shifting from 4.53 to 4.34 ppm on reaction from monomer to polymer. The final copolymers are denoted as $PHMA_nTFEMA_m$, where n and m are the molar percentages for HMA and TFEMA, respectively.

Table S1. Polymer nomenclature, compositions and conversions

Polymer	[HMA]:[TFEMA] (mol L^{-1})	Time (h)	Conversion (%)	^{1}H NMR (ppm)
$PTFEMA_{100}$	0:1	18	93.5%	(400MHz, $CDCl_3$): δ 4.34 (br), 2.01 (br, m), 1.85 (s), 1.57 (s), 1.26 (m), 1.09 (br), 0.94 (br)
$PHMA_{33}TFEMA_{67}$	0.67:0.33	18	92.8%	(400MHz, $CDCl_3$): δ 4.33, 3.94, 1.91 (br, m), 1.61 (br, m), 1.32 (br, m), 1.09 (br), 1.04 (br), 0.90 (br, m)
$PHMA_{50}TFEMA_{50}$	0.5:0.5	18.5	95.1%	(400MHz, $CDCl_3$): δ 4.33 (br), 3.93 (br), 1.89 (br, m), 1.62 (br, m), 1.33 (br), 1.08 (br), 1.02 (br), 0.89 (br, m)
$PHMA_{60}TFEMA_{40}$	0.6:0.4	18.5	95.0%	(60MHz, $CDCl_3$): δ 4.35 (br), 3.95 (br), 2.42 (br, m), 1.94 (br), 1.34 (br), 0.97 (br), 0.91 (br)
$PHMA_{67}TFEMA_{33}$	0.67:0.33	20	87.8%	(400MHz, $CDCl_3$): δ 4.33 (br), 3.93 (br), 1.90 (br), 1.64 (br, m), 1.32 (br, m), 1.07 (br), 1.02 (br), 0.89 (br, m)
$PHMA_{80}TFEMA_{20}$	0.8:0.2	20	93.6%	(60MHz, $CDCl_3$): δ 4.26 (br), 3.96 (br), 1.95 (br), 1.66 (br), 1.35 (br), 0.99 (br), 0.93 (br)
$PHMA_{90}TFEMA_{10}$	0.9:0.1	20	87.4%	(60MHz, $CDCl_3$): δ 4.22 (br), 3.92 (br), 1.92 (br), 1.61 (br), 1.32 (br), 0.96 (br), 0.89 (br)
$PHMA_{100}$	1:0	20	89.1%	(400MHz, $CDCl_3$): δ 3.91 (br), 1.87 (br), 1.60 (br), 1.31 (br), 1.01 (br), 0.87 (br, m)

1.2.5 Example reduction of thiocarbonate chain end

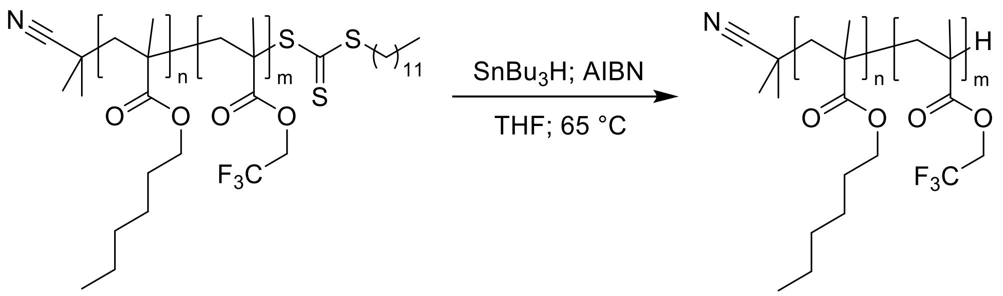

Figure S3. Schematic route to reduce the thiocarbonate end-chain

To an oven-dried 100 mL Young's tap ampoule was added poly(*n*-hexyl methacrylate-*co*-2,2,2-trifluoroethyl methacrylate) (2.00 g; 200 μmol), and 2,2'-azobis(2-methylpropionitrile) (65.7 mg; 400 μmol) these were dissolved in anhydrous tetrahydrofuran (30 mL) before the addition of tri-*n*-butyltin hydride (2.15 mL; 8.0 mmol). Reaction solution was deoxygenated *via* repeated freeze-pump-thaw cycles, before being sealed under a vacuum and heated to 65 °C for 18 hours. Reaction was cooled to room temperature and the solvent removed under reduced pressure *via* a liquid nitrogen-cooled pre-trap. The resulting polymer was purified *via* column chromatography using a 9:1 mixture of silica and potassium carbonate to remove remaining organotin compounds. The crude material was redissolved in dichloromethane and stirred over silver carbonate on celite for 18 hours, filtered and stirred over activated charcoal before being filtered. The resulting solution was washed with 1M aqueous sodium hydroxide, dried, and the solvent removed under reduced pressure. Following reduction, the polymer solution turns from yellow to colourless (Figure S4).

SAFETY NOTE – Organotin compounds are extremely toxic and should be handled with extreme care, all contaminated glassware should be quenched for 24 hours in an aqueous bleach bath.

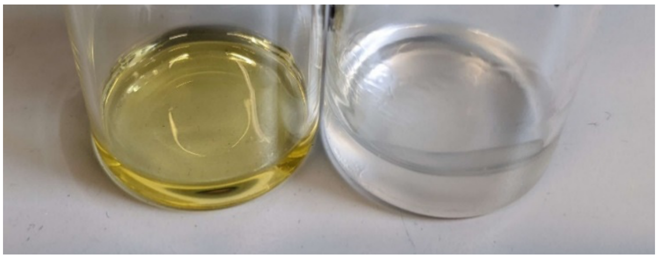

Figure S4. Photograph of poly(hexyl methacrylate-co-trifluoroethyl methacrylate) in THF after initial polymerisation step (left) and after post-polymerisation reduction (right).

Table 2. Reaction yields and 1H NMR assignments for PHMAnTFEMAm after end-group reduction.

Polymer	Yield (%)	^{1}H NMR (ppm)
$PTFEMA_{100}$	73.1 %	(60MHz, $CDCl_3$): δ 4.29 (br, m), 1.95 (br), 1.73 (br), 1.26 (br), 1.09 (br), 0.97 (br)
$PHMA_{33}TFEMA_{67}$	69.8 %	(60MHz, $CDCl_3$): δ 4.27 (br, m), 3.96 (br, m), 1.94 (br), 1.69 (br), 1.31 (br), 0.98 (br), 0.91 (br)
$PHMA_{50}TFEMA_{50}$	79.4 %	(60MHz, $CDCl_3$): δ 4.27 (br, m), 3.95 (br, m), 1.93 (br), 1.62 (br), 1.34 (br), 0.98 (br), 0.91 (br)
$PHMA_{60}TFEMA_{40}$	77.5%	(60MHz, $CDCl_3$): δ 4.39 (br, m), 3.95 (br, m), 1.93 (br), 1.80 (br), 1.33 (br), 0.97 (br), 0.90 (br)
$PHMA_{67}TFEMA_{33}$	68.0 %	(60MHz, $CDCl_3$): δ 4.23 (br, m), 4.11 (br, m), 1.93 (br), 1.73 (br), 1.31 (br), 0.94 (br), 0.89 (br)
$PHMA_{80}TFEMA_{20}$	72.7 %	(60MHz, $CDCl_3$): δ 4.24 (br, m), 3.94 (br, m), 1.94 (br, m), 1.74 (br), 1.33 (br), 0.90 (br)
$PHMA_{90}TFEMA_{10}$	80.2 %	(60MHz, $CDCl_3$): δ 4.34 (br, m), 3.93 (br, m), 1.90 (br), 1.73 (br), 1.32 (br), 0.90 (br)
$PHMA_{100}$	76.2 %	(60MHz, $CDCl_3$): δ 3.95 (br, m), 1.94 (br), 1.74 (br), 1.33 (br), 0.98 (br), 0.91 (br)

1.3 Preparation of polymer films

Films were drop-cast onto a 12 mm diameter circular glass coverslip using an aliquot (25 μL) of a solution of the polymer in DCM (200 mg ml^{-1}), covered with aluminium foil and allowed to evaporate overnight, yielding thick films (~ 200 μm) thick. The final films were covered with a second glass coverslip to aid handling during measurement.

PdOEP:DPA doped films were prepared in the same way, by first mixing 100 μL of polymer stock solution with 20 μL of a PdOEP in DCM solution (0.3 mM) and 20 μL of a DPA in DCM solution (30 mM), before casting. DPA-only and PdOEP-only films were prepared in the same way (i.e., without PdOEP and DPA addition, respectively).

2 Instrumentation

2.1 Nuclear magnetic resonance (NMR) spectroscopy

^{1}H and ^{13}C nuclear magnetic resonance spectra were recorded on a Bruker Avance III 400 or Magritek Spinsolve 60 spectrometer at 293 K. Chemical shifts are reported as δ in parts per million (ppm) and referenced to the chemical shift of the residual solvent resonances ($CDCl_3$: ^{1}H: δ = 7.26 ppm, ^{13}C: δ = 77.16 ppm).

2.2 Size-exclusion chromatography (SEC)

Polymer molecular weight and dispersity were determined using a Malvern Viscotek GPCmax size exclusion chromatograph instrument fitted with a Viscotek TDA 305 detector unit equipped with refractive index and light scattering detectors. Samples were dissolved in tetrahydrofuran at a concentration of approximately 1 mg mL^{-1} and eluted through a guard column and two Agilent PLGel 5 µm mixed C columns (300 × 7.5 mm) at a flow rate of 1 $ml.min^{-1}$; the elution pathlength was heated to 30 °C for the duration. Molecular weights were calibrated against known poly(methyl acrylate) standards.

2.3 Differential scanning calorimetry (DSC)

Differential scanning calorimetry was conducted using a TA Instruments Discovery 2500. Samples were analysed in non-hermetic aluminium pans and compared against an empty reference pan of the same type. Loaded sample masses were between 3 and 10 mg. Samples were subjected to two complete heat/cool cycles from -50 °C to 150 °C (-85 °C to 150 °C for lower T_g samples) and both heating and cooling rates were set at 10 °C min^{-1}

2.4 UV/vis transmittance and absorption spectroscopy

UV/Vis transmittance and absorption spectra were measured with a PerkinElmer Lambda 750 spectrophotometer. Transmittance spectra of films were measured using wavelength scan with a resolution of 1 nm at a scan speed of 267 nm/min and a slit width of 2 nm. Samples were directly mounted to the sample holder.

Solution spectroscopy was carried out on solutions in THF in quartz SUPRASIL® cuvettes (10 mm pathlength). Absorption spectra of luminophore solutions were taken using a wavelength scan with a resolution of 0.5 nm at a scan speed of 141.20 nm/min and a slit width of 2 nm. A reference sample of THF in an identical cuvette was used to apply a 100% transmission correction.

2.5 Steady-state photoluminescence (PL) spectroscopy

Steady-state PL spectroscopy was performed on a Fluorolog-3 spectrophotometer (Horiba Jobin Yvon). Solid-state emission spectra were recorded using the front-face configuration. Solution emission spectra were recorded using the right-angle configuration, over 10 averaged scans. The excitation and emission slits were adjusted so that the maximum PL intensity was within the range of linear response of the detector and were kept the same between samples if direct comparison between the emission intensity was required. Emission and excitation spectra were corrected for the wavelength response of the system and the intensity of the lamp profile over the excitation range, respectively, using correction factors supplied by the manufacturer. Photoluminescence quantum yields (Φ_{PL}) were measured using a Quanta-phi integrating sphere (Horiba Jobin Yvon) mounted on the Fluorolog-3 spectrophotometer.

2.6 Upconversion, phosphorescence and time-resolved emission measurements

The UC emission and phosphorescence spectra, threshold intensity (I_{th}), UC quantum yield (Φ_{UC}) and lifetime measurements were performed using an FLS1000 time-correlated single photon counting (TCSPC) spectrometer (Edinburgh Instruments Ltd.). The samples were excited with a 532 nm laser (MGL-III-532, 200mW). To determine I_{th}, the laser power was adjusted using a Thorlabs PM100A Power Meter Console combined with a S120VC Si photodiode power sensor (range: 200-1100 nm) before the measurement, across the 5 to 8000 mW cm^{-2}.

2.6.1 UC quantum yield

The Φ_{UC} was measured with an integrating sphere (SNS125 5-inch sphere, three windows, International Light Technologies, Figure S5). The sample was placed at the center of the sphere using a sample holder. A baffle is placed in front of the observation window, which blocks any scattering and reflection of the laser from the sample surface. The angle of the sample holder is adjustable. The normal direction of the sample holder is 22.5° to the excitation beam line, which leads the reflection of the laser to the inner surface of the sphere.

The laser power was measured with a photodiode before each Φ_{UC} measurement. Both the emission of the sample (380-500 nm) and scattering of the laser beam (530-534 nm) were measured. A neutral density filter (O.D.=3.0) was placed before the excitation beam for the scattering intensity measurements. Six data sets were collected to calculate the Φ_{UC} of each sample: 1. sample in the path of the beam – "in fluorescence"; 2. sample in scattering; 3. sample

facing away from beam – "out of fluorescence", 4. sample out of scattering; 5. empty sphere fluorescence; 6. empty sphere scattering.

Three sets of data were collected for each TTA-UC doped sample. The parallel data sets were calculated separately, which gives three Φ_{UC} results for each sample, and the reported Φ_{UC} is the average of these data, along with the standard deviation of the measurements. During the 'sample in beam' measurement, the sample was facing toward both the excitation window and the observation window, while in the sample-out mode, the holder was turned 180° to have the back of the holder facing the windows. For the fluorescence measurement (Data sets 1 and 3), the bandwidth was 1 nm for the detector, and the scan step was 1 nm per data point with duration of 1 second, scanned from 380 nm to 500 nm. For the scattering measurement (Data sets 2 and 4), the bandwidth was 1 nm for the detector, and the scan step was 0.1 nm per data point with duration of 0.1 second, scanned from 530 nm to 534 nm. The transmittance of the filter at the excitation wavelength was measured with a UV-Vis absorption spectrometer (DS5, Edinburgh Instruments Ltd.), taking the average over 10 parallel measurements. The empty-sphere data sets (Data sets 5 and 6) were collected at the beginning of the measurement, under the same conditions of the sample-in measurement, which were shared in all calculations of samples measured in the same day. During the calculation, all data were corrected by the transmittance of each filter used, and normalised based on the slit-width, scan step and the scan duration used.

The Φ_{UC} was calculated using the experimental approach described by Porrès *et al.*[2] and the following formulae:[3,4]

$$\Phi_{\mathrm{UC}} = \frac{E_{x,in} - (1-A)E_{x,out}}{AL_{b,in}} \qquad \text{(Equation S1)}$$

where *A* is the percentage of the photons absorbed directly by the sample, which is corrected by removing the secondary absorption from the sphere-reflected photons:

$$A = \frac{L_{x,out} - L_{x,in}}{L_{x,out}} \qquad \text{(Equation S2)}$$

where *E* is the integrated photon counts from emission spectra, and *L* is the integrated photon counts from the scattering spectra. *x* delineates sample, while *b* is blank. *In* means the sample was in the path of the excitation beam, and *out* delineates the sample is out of the beam line. A quantum yield is defined as the ratio of absorbed to emitted photons, meaning the Φ_{UC} is limited to 50% since this is a bimolecular process. While some papers report this as a normalised value, Φ_{UC} is reported to its un-normalised value here, which is capped at 50%.[5]

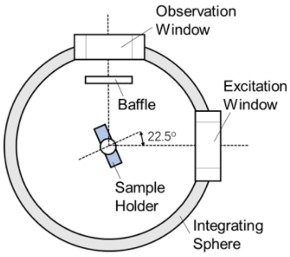

Figure S5. Integrating sphere set-up used to determine Φ_{UC}.

2.6.2 Lifetime measurements

Fluorescence decay measurements were performed using the multi-channel scaling (MCS) method on a the FLS1000 TCSPC spectrometer. The emission decay was recorded using a photomultiplier tube (PMT-980) equipped with TCC2 counting electronics. For the upconversion lifetime measurements, a wavelength of 440 nm was selected, and a short-pass filter (cut-off at 500 nm, Thorlabs) was placed in front of the detector. For the phosphorescence lifetimes, a wavelength of 660 nm was selected, and a long-pass filter (cut-off 550 nm, Thorlabs) was used. The instrument response function (IRF) was measured using Ludox® colloidal silica solution (a SiO_2 particle suspension solution) and using a neutral density filter (O.D.=3) to attenuate the laser intensity. The pulse repetition rate was adjusted to ensure the full decay was detected within the time window. Data-fitting was carried out by tail fitting to each emission decay trace using a multiexponential decay function within the FAST software package (Edinburgh Instruments Ltd.). The goodness of fit was evaluated using the reduced chi-square statistics (χ^2) and the randomness of the residuals.[6] Based on the lifetimes (τ_i) and their fractional contributions (f_i), the average lifetime ($<\tau>$) was calculated to facilitate a better comparison of the emission decay across different samples. The $<\tau>$ was obtained using the following equation:

$$<\tau> = f_1\tau_1 + f_2\tau_2 \qquad \text{(Equation S3)}$$

3 Supporting Experimental Data

3.1 Nuclear Magnetic Resonance Spectra

3.1.1 Chain Transfer Agent Synthesis

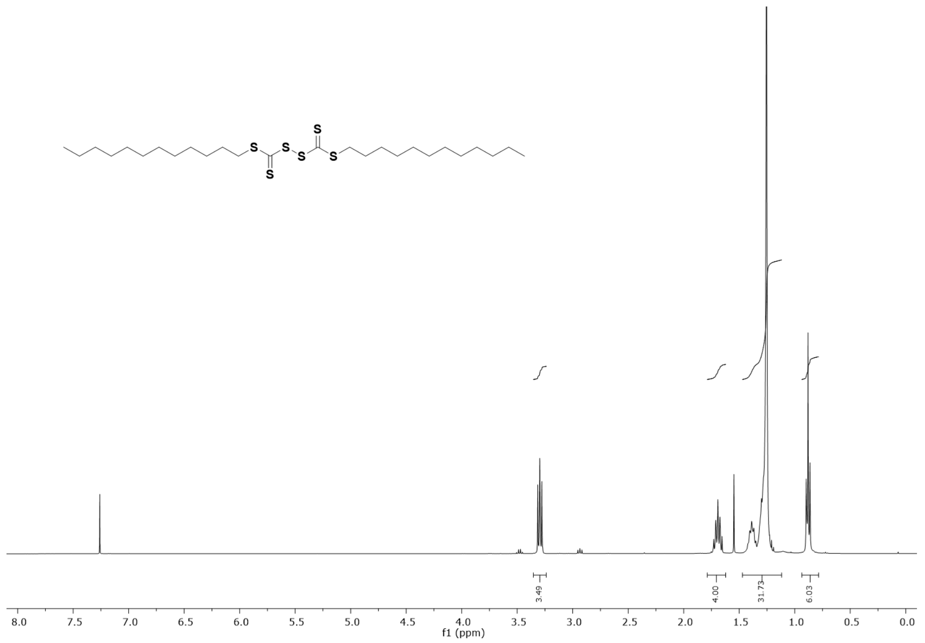

Figure S6. ^{1}H NMR (400 MHz) spectrum of bis(dodecyl trithiocarbonate).

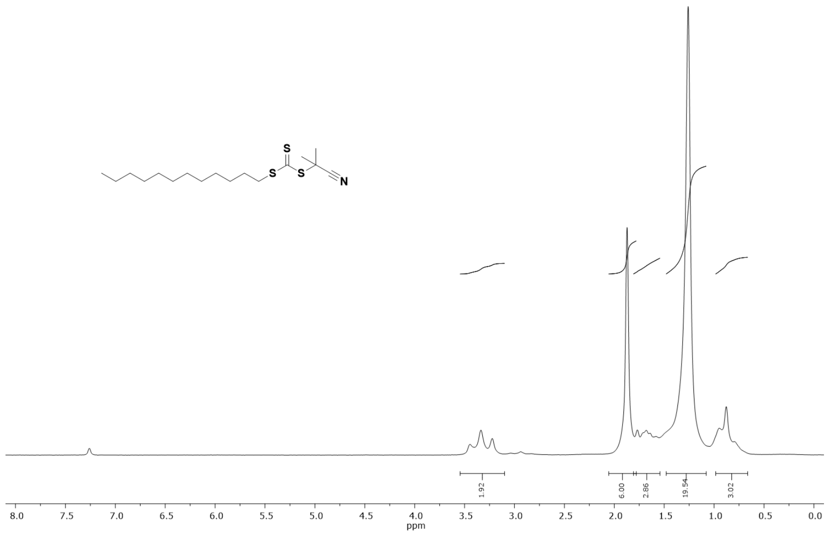

Figure S7. ^{1}H NMR (60 MHz) spectrum of 2-cyanopropan-2-yl dodecyl trithiocarbonate.

3.2 Polymer Synthesis

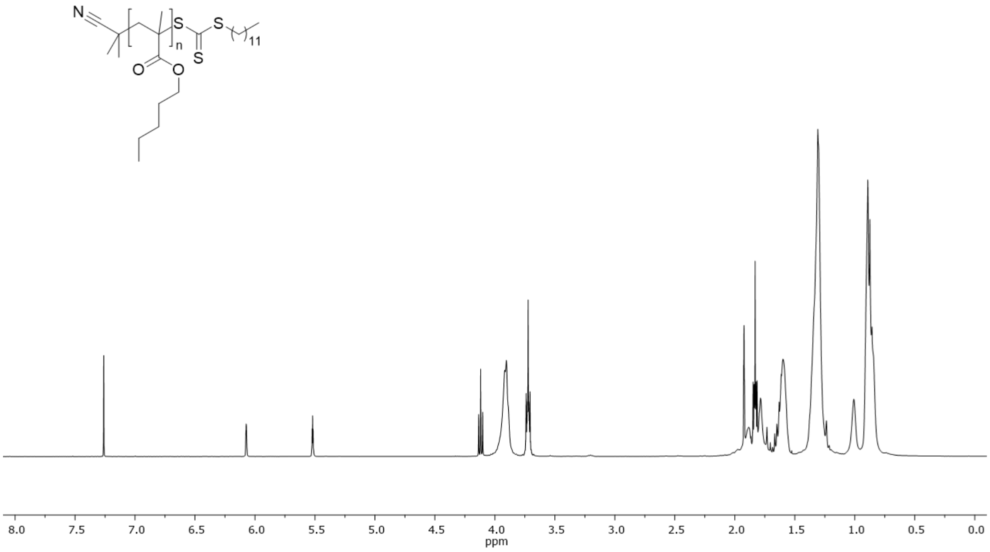

Figure S8. ^{1}H NMR (400 MHz) spectrum of $PHMA_{100}$

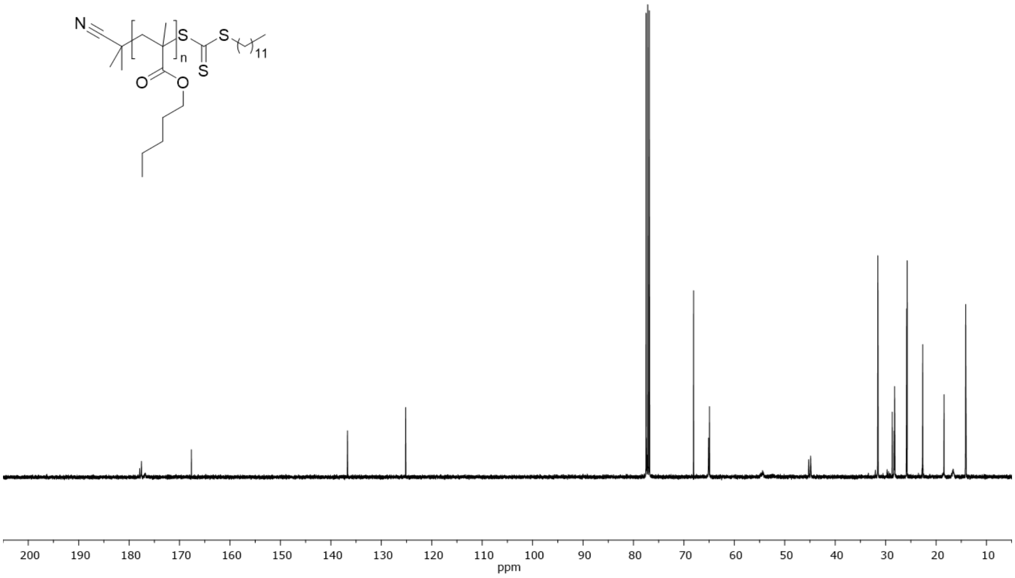

Figure S9. ^{13}C NMR (400 MHz) spectrum of $PHMA_{100}$

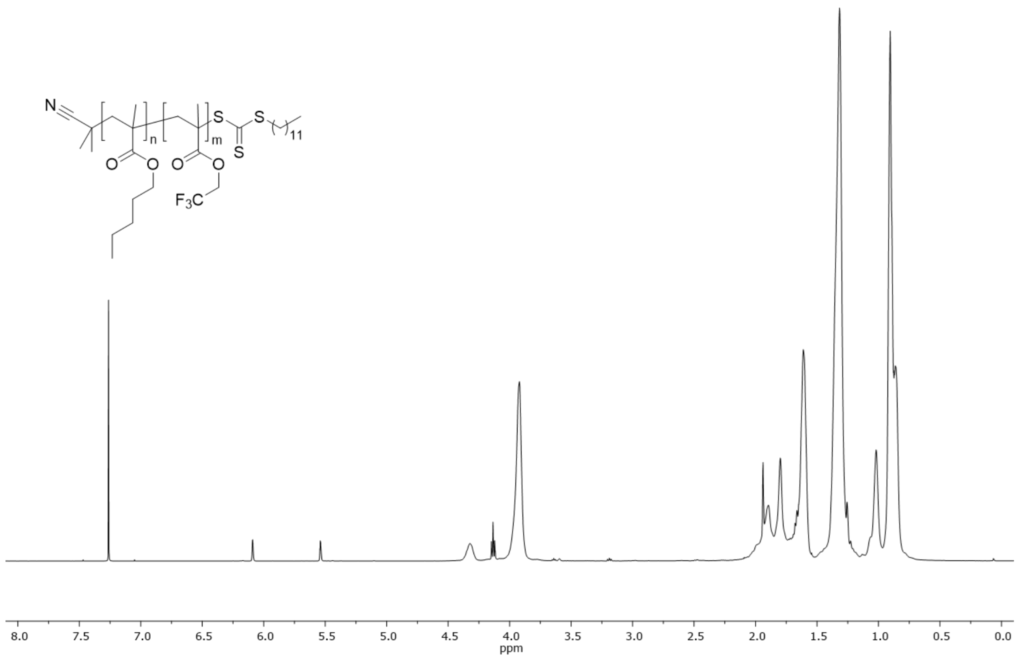

Figure S10. ^{1}H NMR (400 MHz) spectrum of $PHMA_{90}TFEMA_{10}$

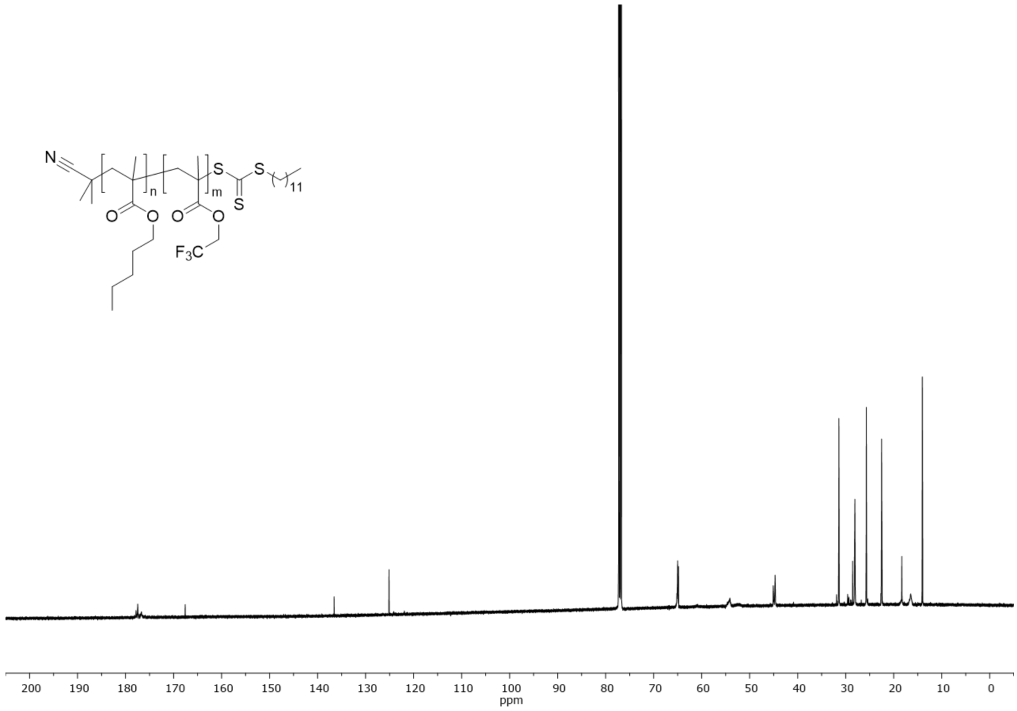

Figure S11. ^{13}C NMR (400 MHz) spectrum of $PHMA_{90}TFEMA_{10}$

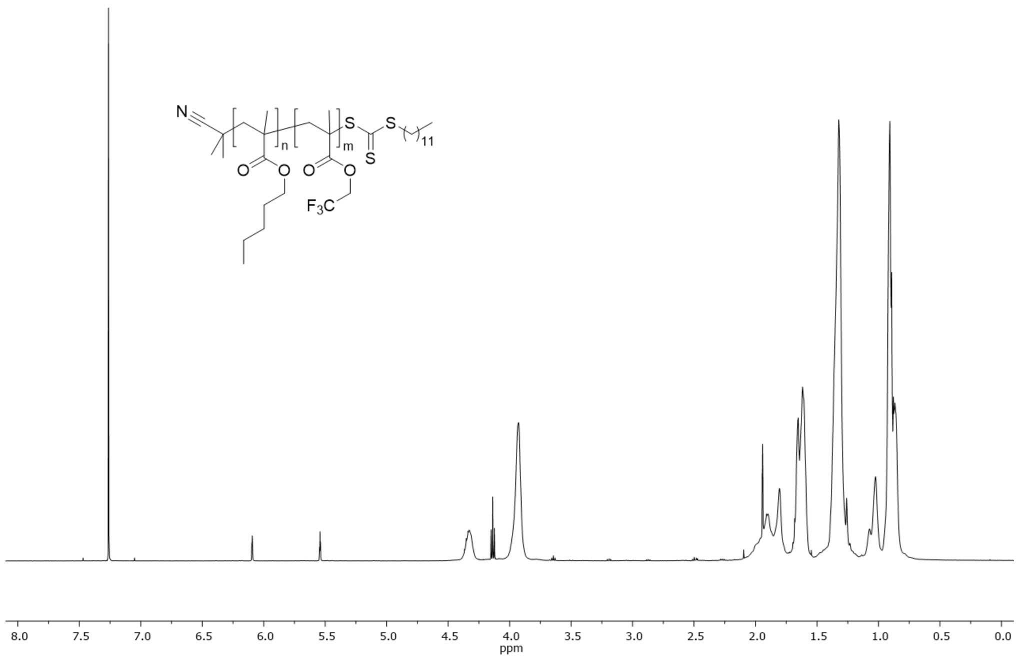

Figure S12. ^{1}H NMR (400 MHz) spectrum of $PHMA_{80}TFEMA_{20}$

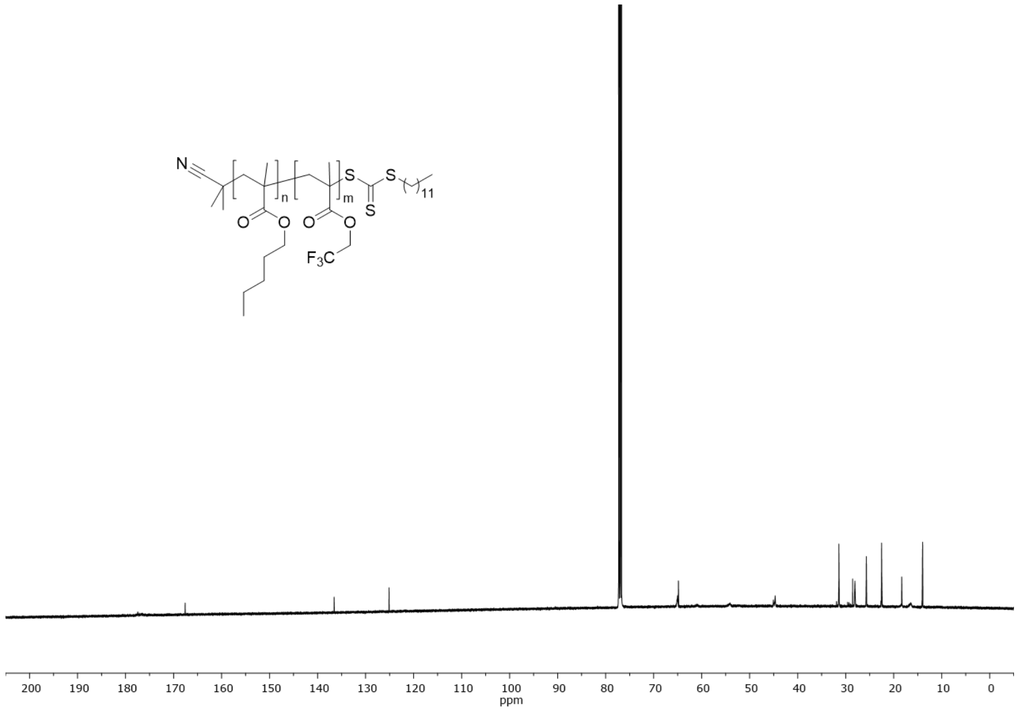

Figure S13. ^{13}C NMR (400MHz) spectrum of $PHMA_{80}TFEMA_{20}$

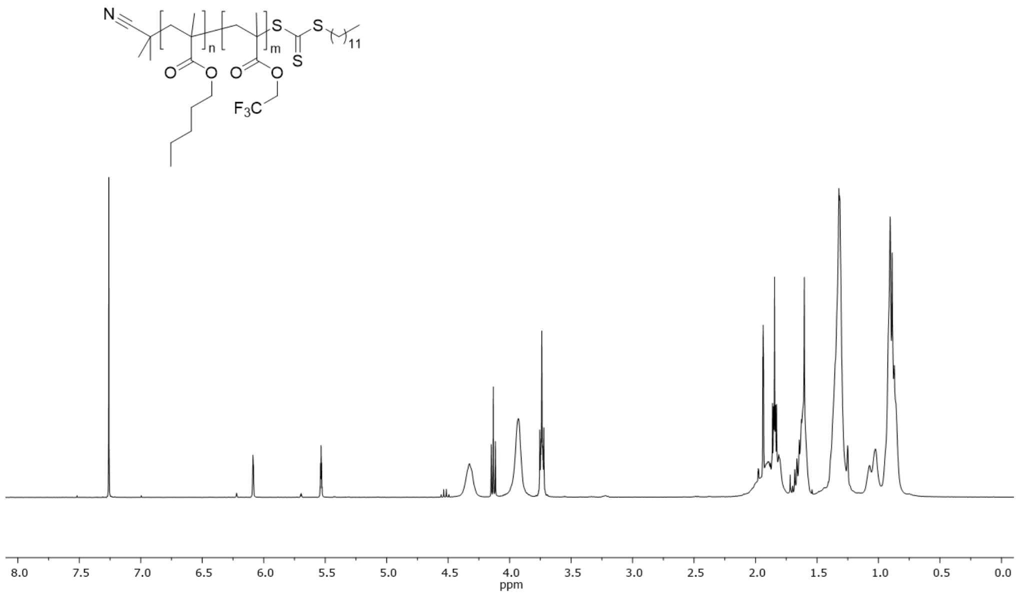

Figure S14. ^{1}H NMR (400 MHz) spectrum of $PHMA_{67}TFEMA_{33}$

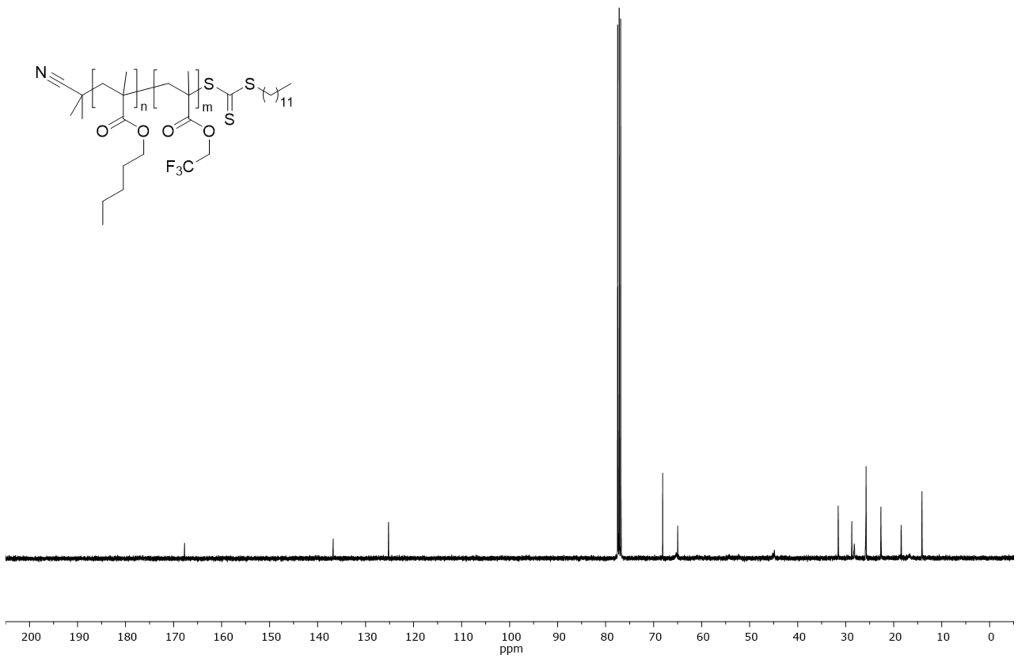

Figure S15. ^{13}C NMR (400 MHz) spectrum of $PHMA_{67}TFEMA_{33}$

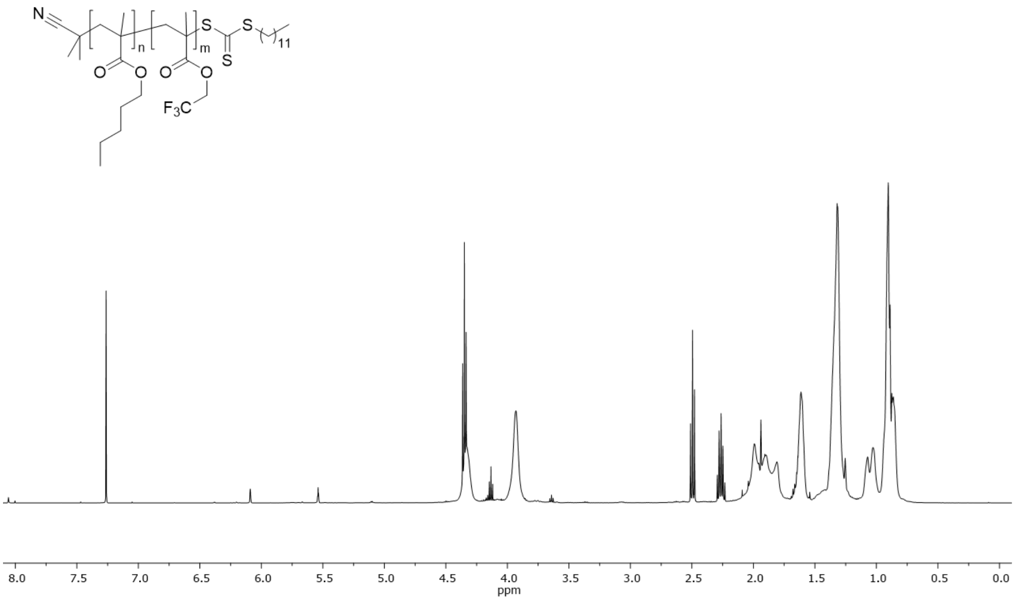

Figure S16. ^{1}H NMR (400 MHz) spectrum of $PHMA_{60}TFEMA_{40}$

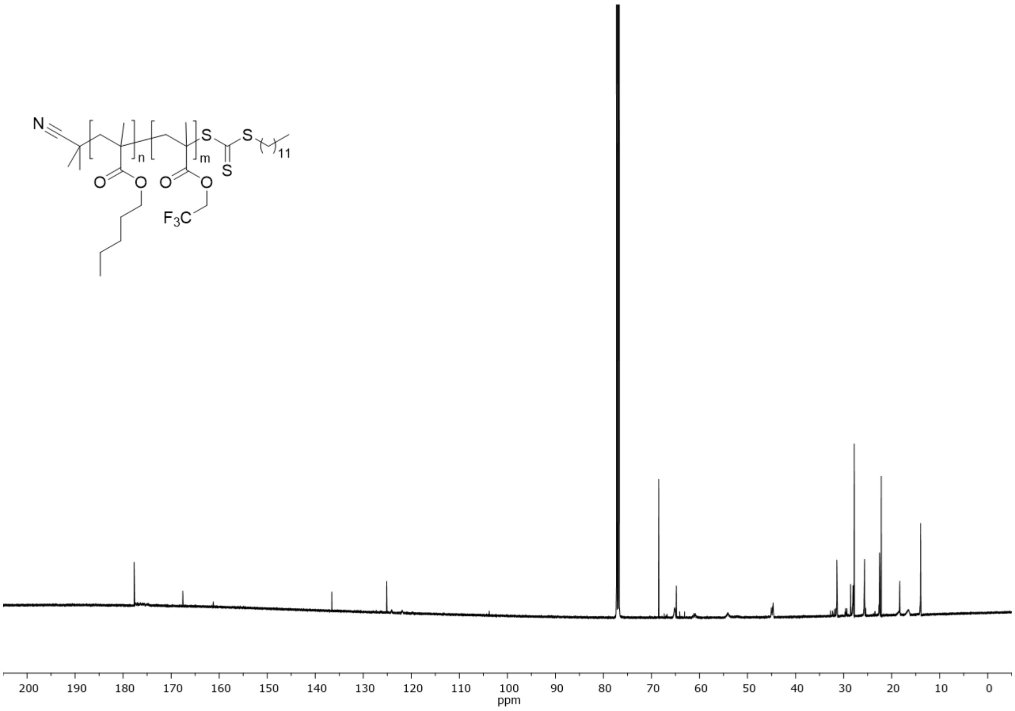

Figure S17. ^{13}C NMR (400 MHz) spectrum of $PHMA_{60}TFEMA_{40}$

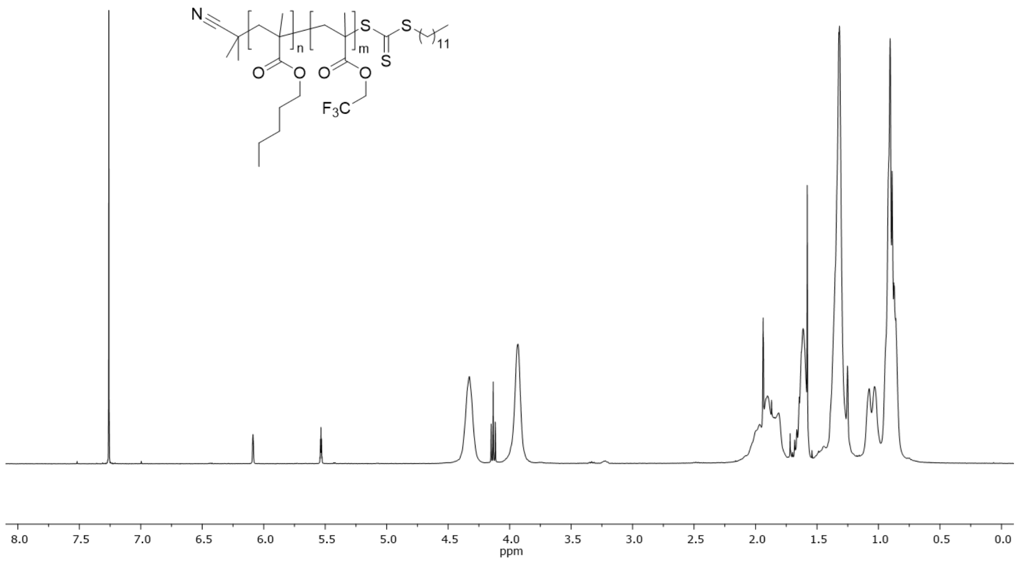

Figure S18. ^{1}H NMR (400 MHz) spectrum of $PHMA_{50}TFEMA_{50}$.

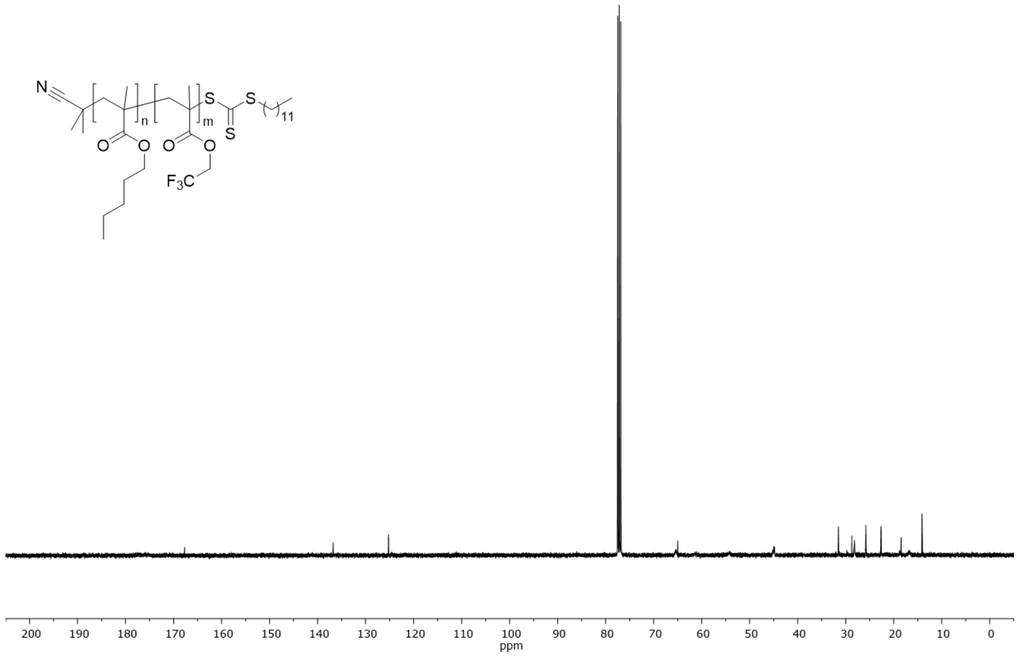

Figure S19. ^{13}C NMR (400 MHz) spectrum of $PHMA_{50}TFEMA_{50}$

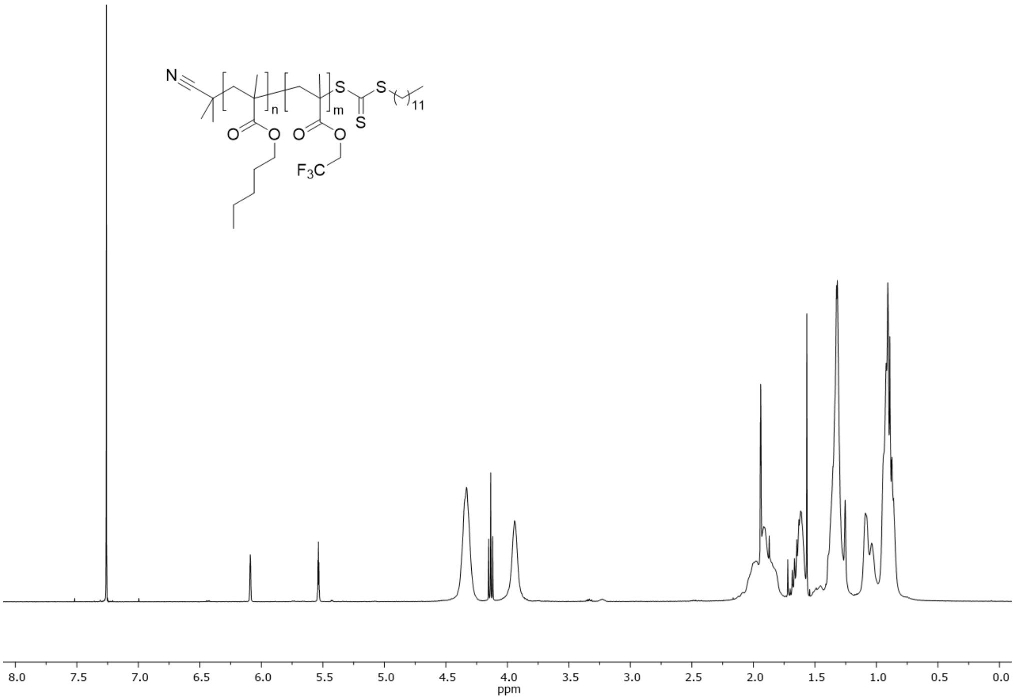

Figure S20. ^{1}H NMR (400 MHz) spectrum of $PHMA_{33}TFEMA_{67}$

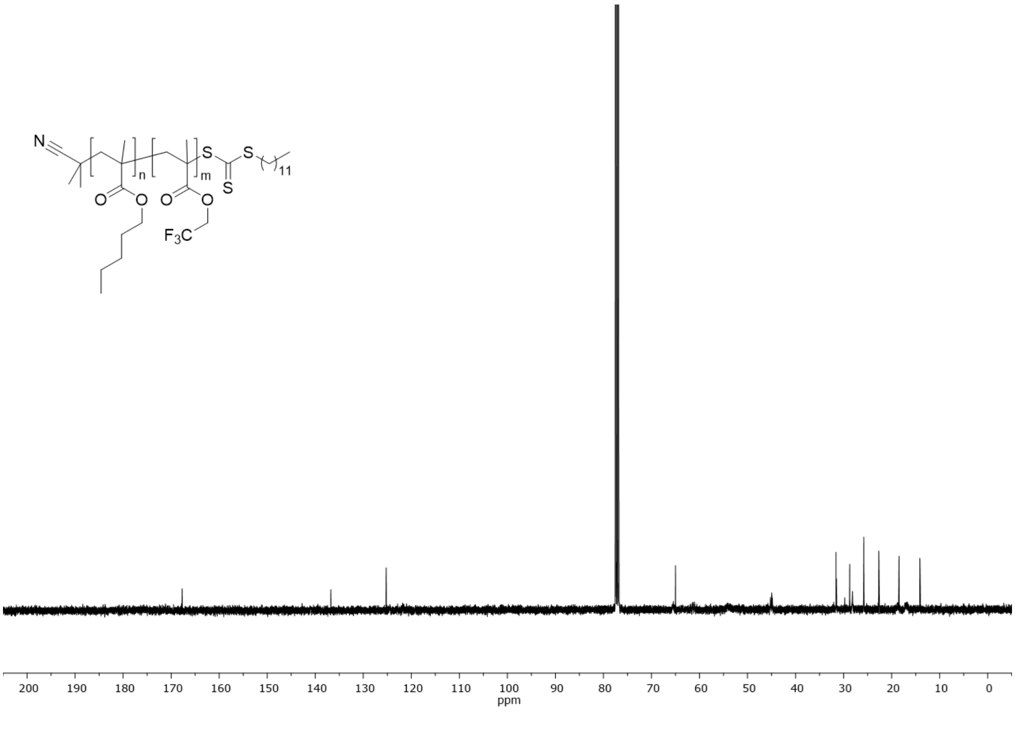

Figure S21. ^{13}C NMR (400 MHz) spectrum of $PHMA_{33}TFEMA_{67}$

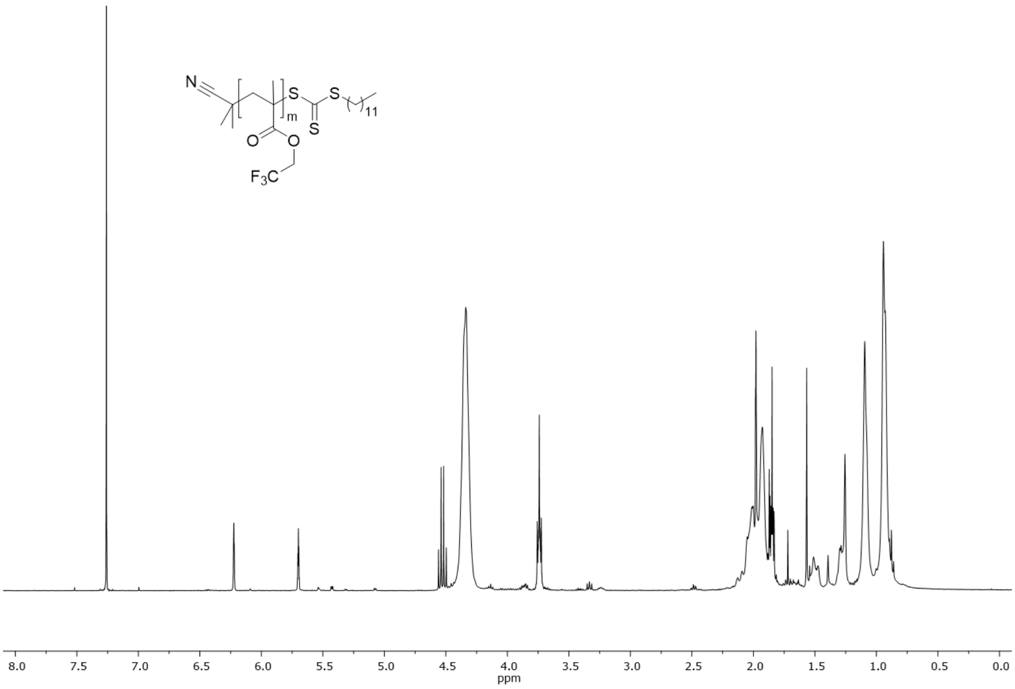

Figure S22. ^{1}H NMR (400 MHz) spectrum of $PTFEMA_{100}$

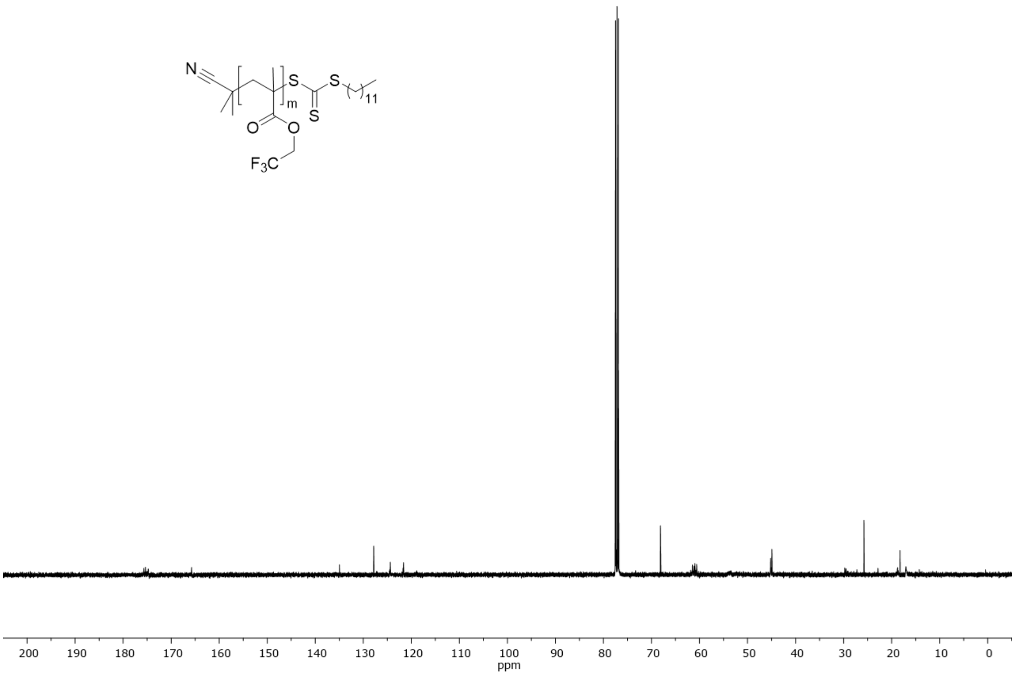

Figure S23. ^{13}C NMR (400 MHz) spectrum of $PTFEMA_{100}$.

1.1 Chain-end reduction

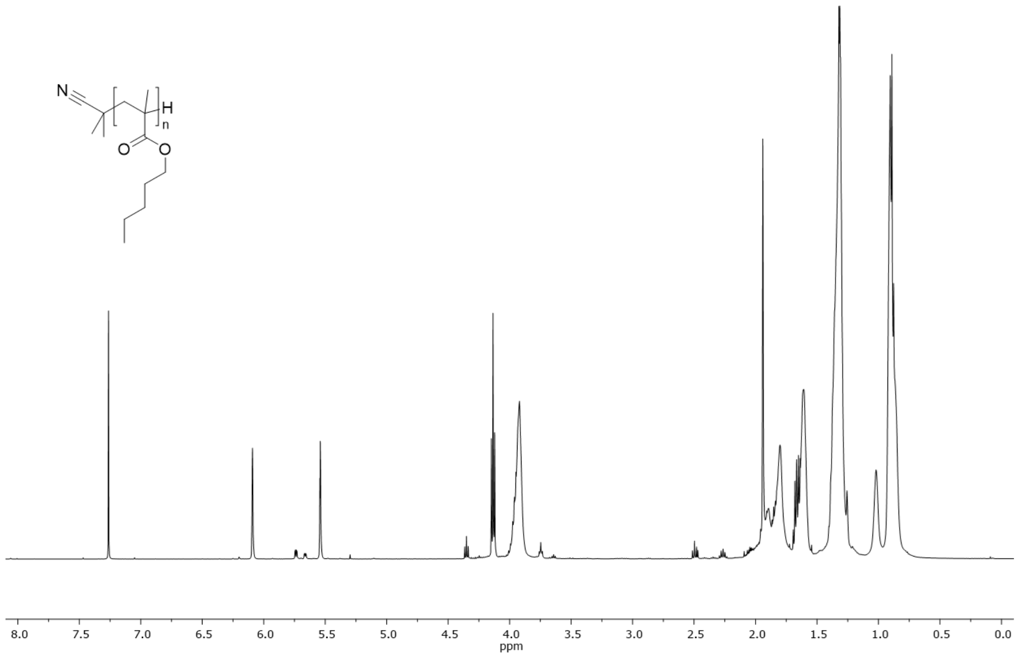

Figure S24. ^{1}H NMR (400 MHz) spectrum of end-reduced $PHMA_{100}$.

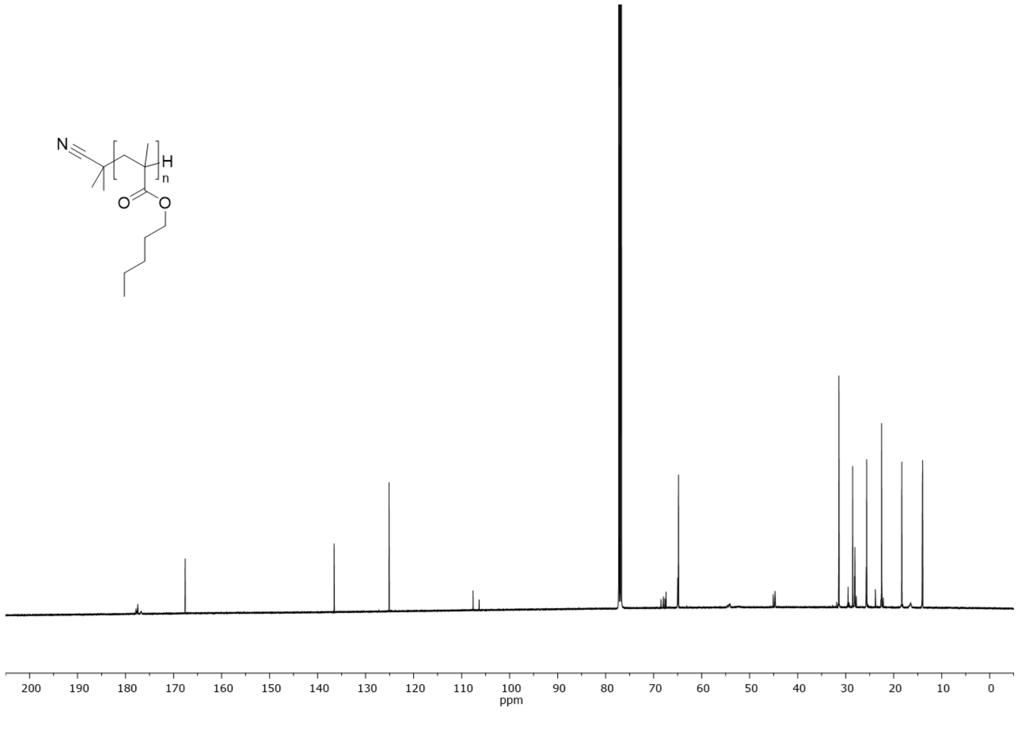

Figure S25. ^{13}C NMR (400 MHz) spectrum of end-reduced $PHMA_{100}$

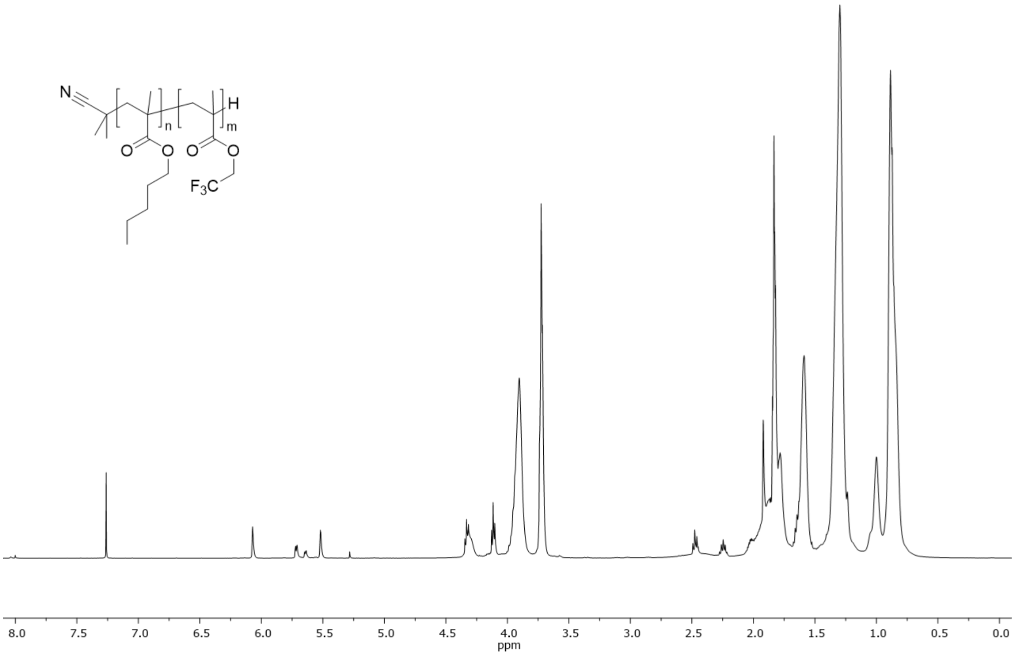

Figure S26. ^{1}H NMR (400 MHz) spectrum of end-reduced $PHMA_{90}TFEMA_{10}$.

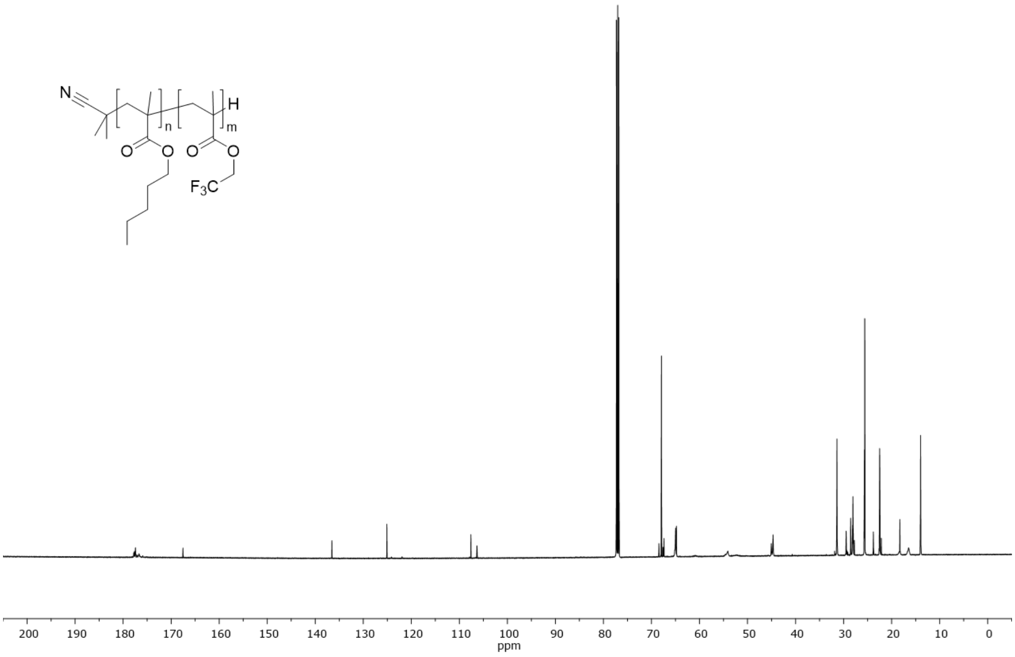

Figure 27. ^{13}C NMR (400 MHz) spectrum of end-reduced $PHMA_{90}TFEMA_{10}$.

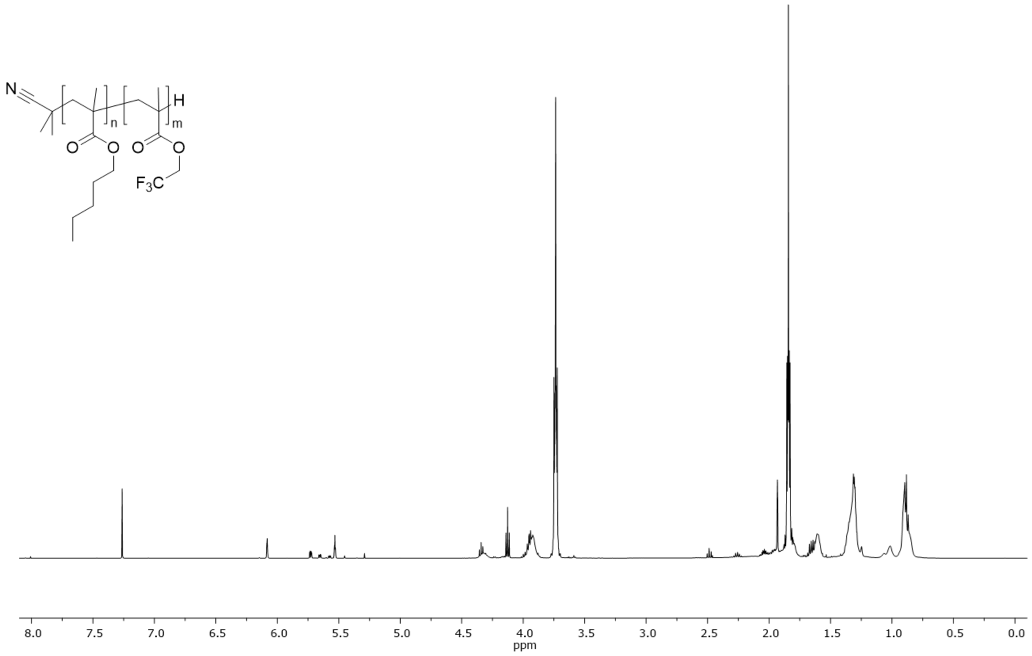

Figure S28. ^{1}H NMR (400 MHz) spectrum of end-reduced $PHMA_{80}TFEMA_{20}$.

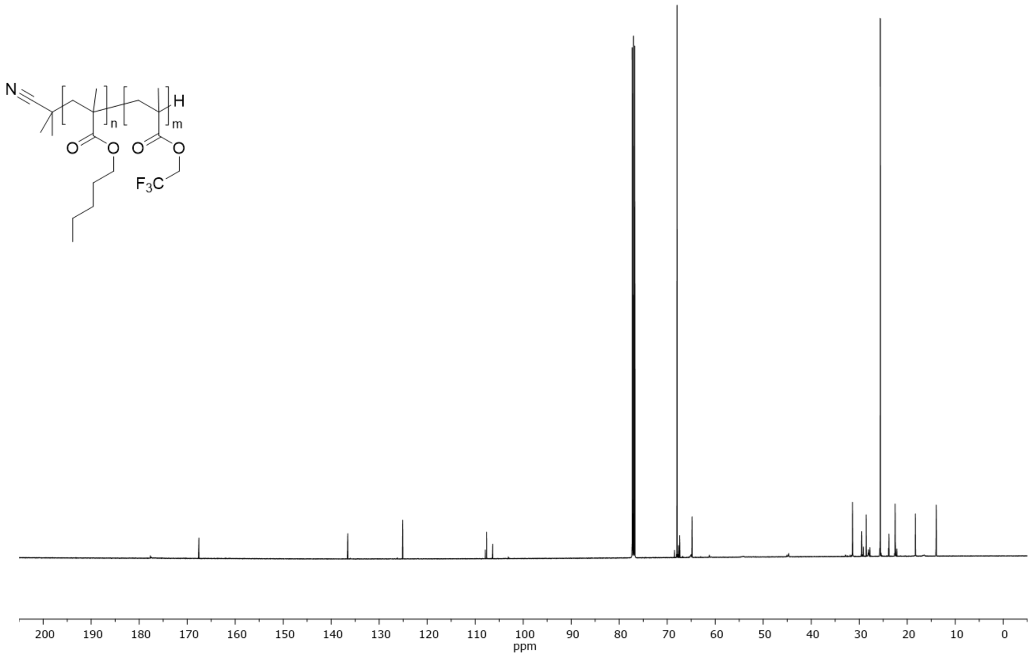

Figure S29. ^{13}C NMR (400 MHz) spectrum of end-reduced $PHMA_{80}TFEMA_{20}$.

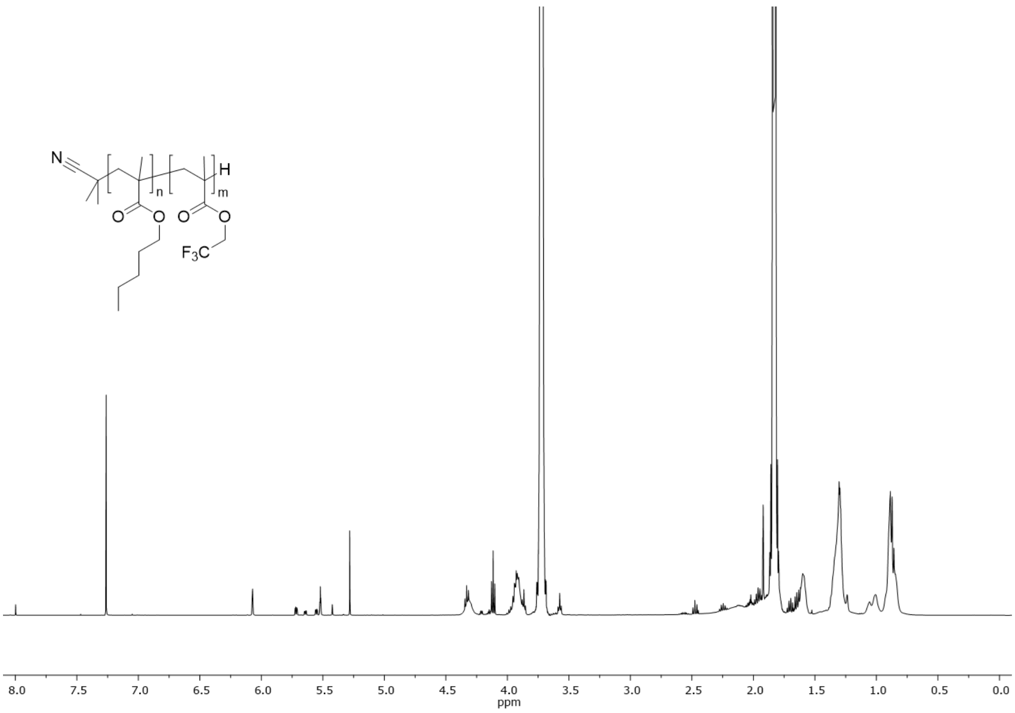

Figure S30. ^{1}H NMR (400 MHz) spectrum of end-reduced $PHMA_{67}TFEMA_{33}$.

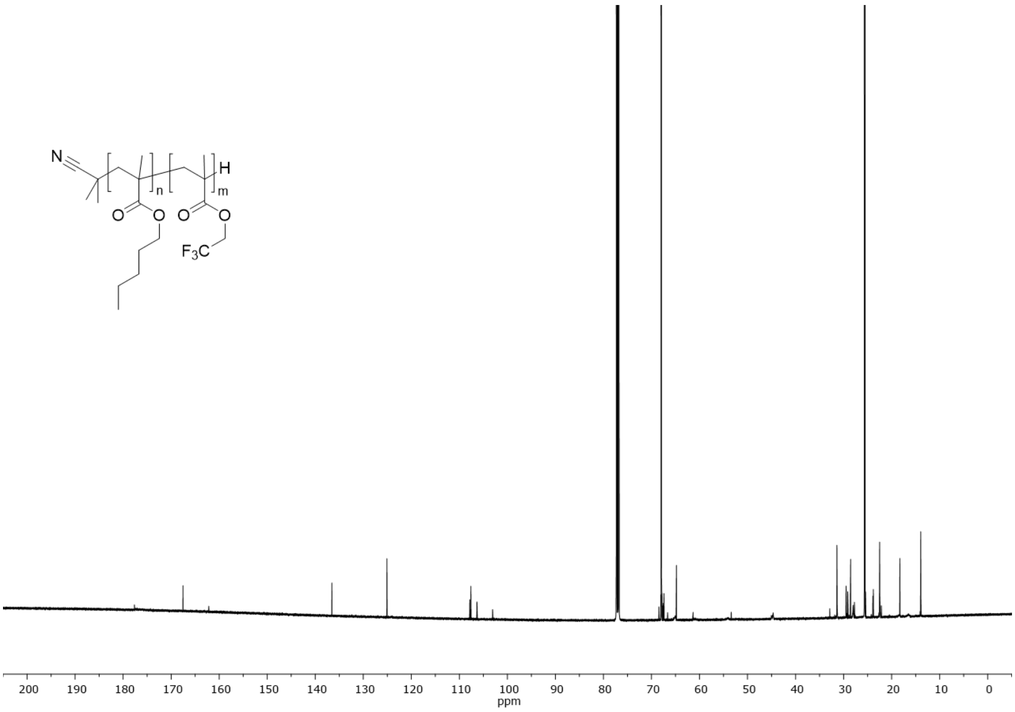

Figure S31. ^{13}C NMR (400 MHz) spectrum of end-reduced $PHMA_{67}TFEMA_{33}$.

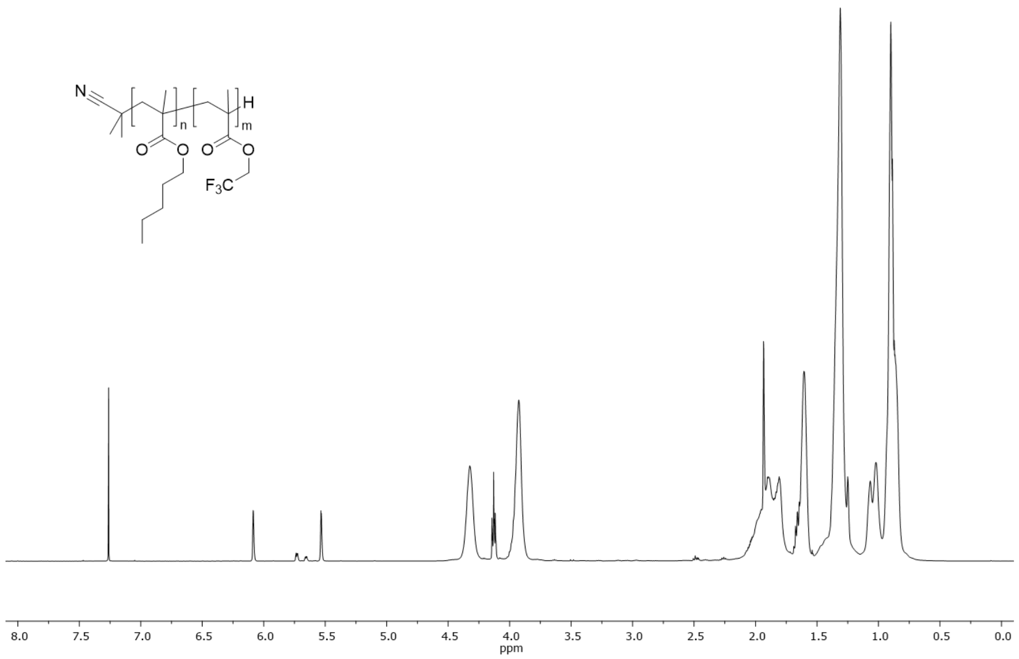

Figure S32. ^{1}H NMR (400 MHz) spectrum of end-reduced $PHMA_{60}TFEMA_{40}$.

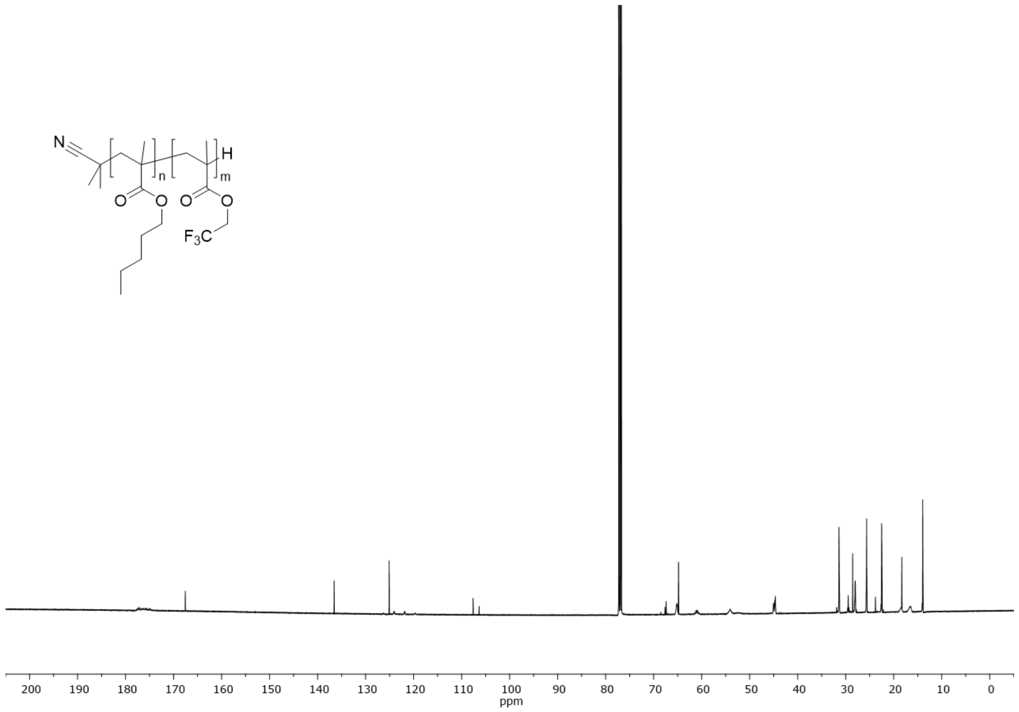

Figure S33. ^{13}C NMR (400 MHz) spectrum of end-reduced $PHMA_{60}TFEMA_{40}$.

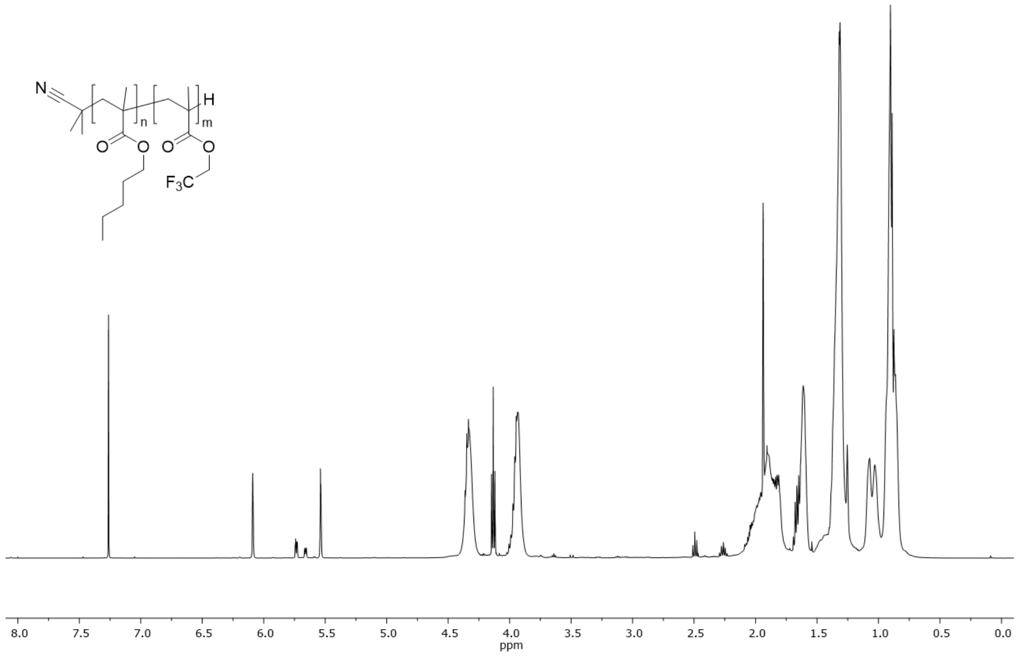

Figure S34. ^{1}H NMR (400 MHz) spectrum of end-reduced $PHMA_{50}TFEMA_{50}$.

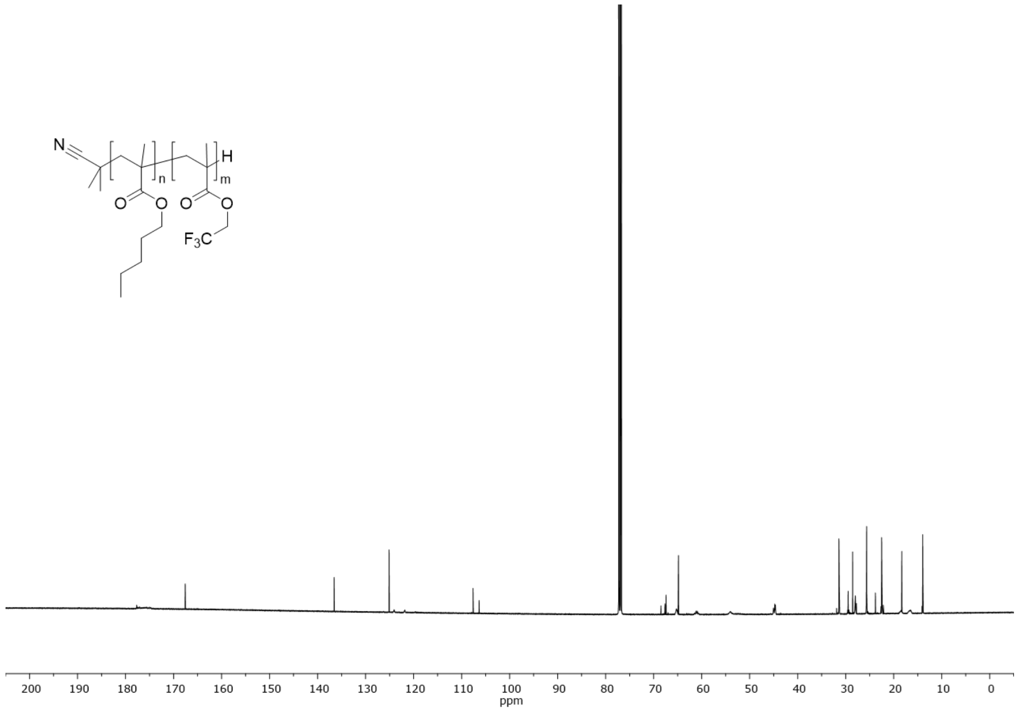

Figure S35. ^{13}C NMR (400 MHz) spectrum of end-reduced $PHMA_{50}TFEMA_{50}$.

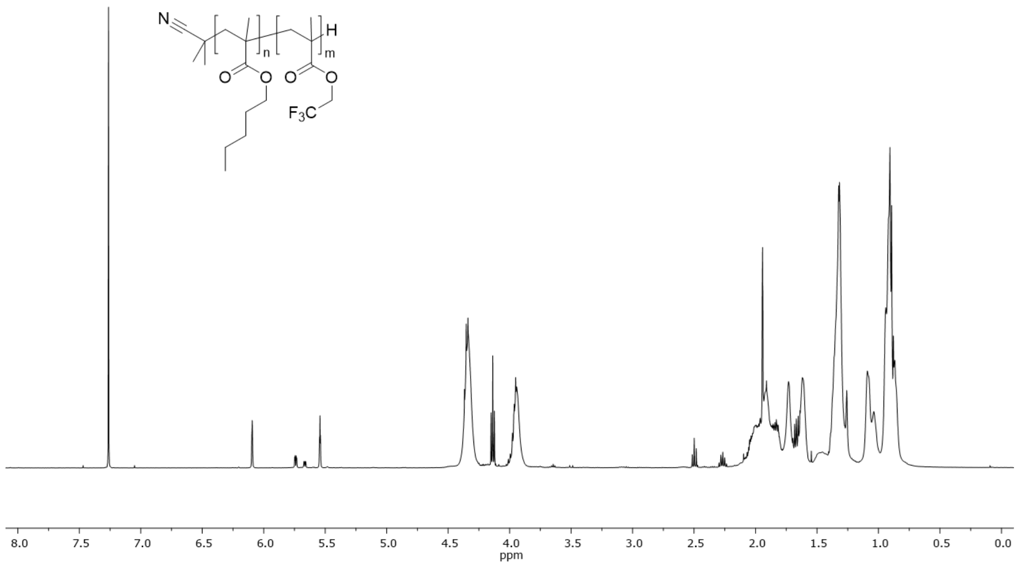

Figure S36. ^{1}H NMR (400 MHz) spectrum of end-reduced $PHMA_{33}TFEMA_{67}$.

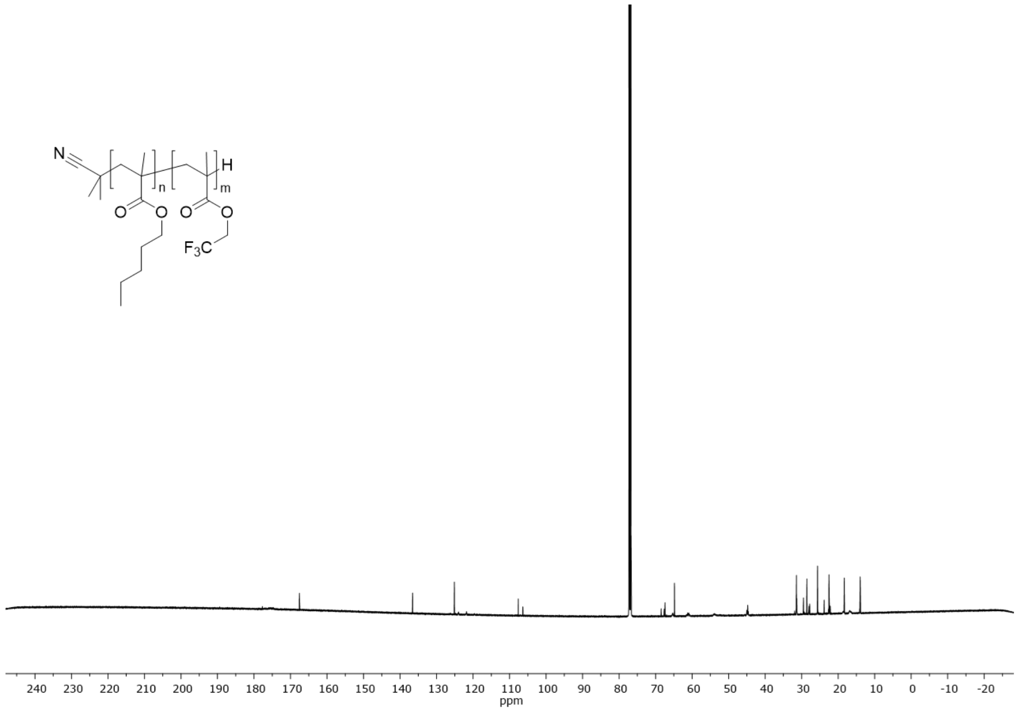

Figure S37. ^{13}C NMR (400 MHz) spectrum of end-reduced $PHMA_{33}TFEMA_{67}$.

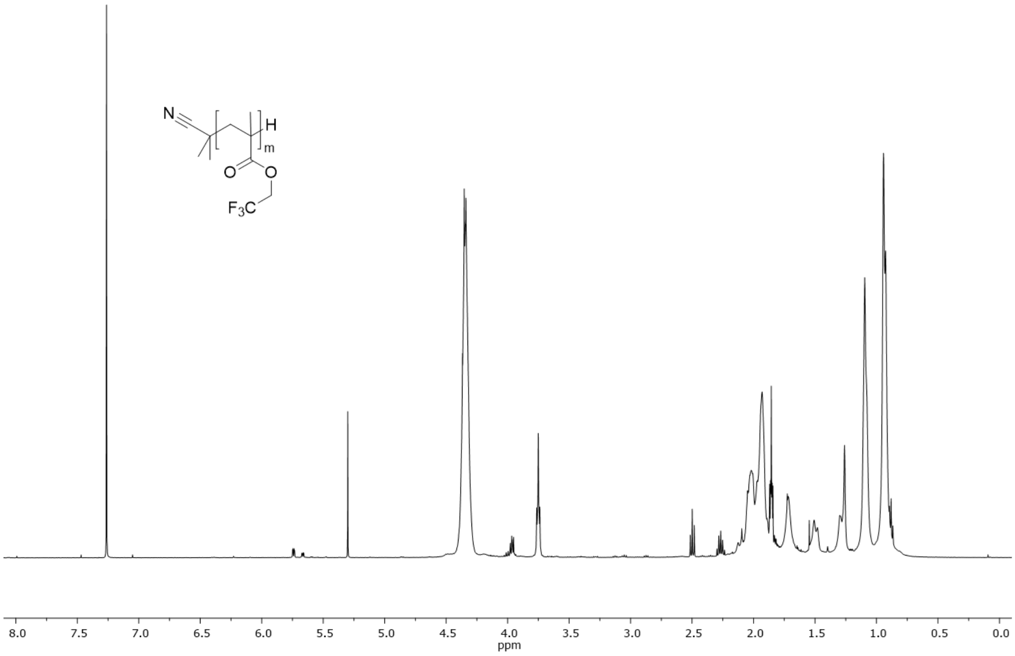

Figure S38. ^{1}H NMR (400 MHz) spectrum of end-reduced $PTFEMA_{100}$.

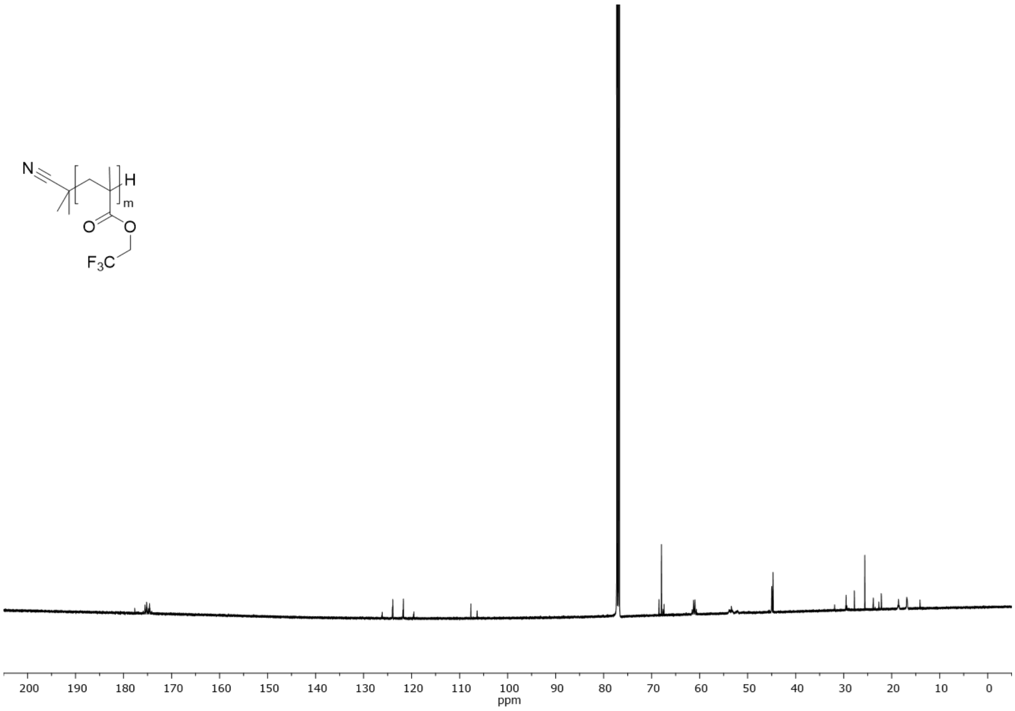

Figure S39. ^{13}C NMR (400 MHz) spectrum of end-reduced $PTFEMA_{100}$.

3.3 Molecular weight determination

The theoretical number-average molecular weight (M_n) for each polymer was calculated based on the sum of the targeted degree of polymerisation for each monomer, with the addition of the CTA molecular weight.

The actual polymer molecular weights (number- and weight-average, (M_w) and dispersity (Đ) were characterised by SEC, as summarised in Table S3.

Table S3. Theoretical and measured molecular weights for synthesised polymers.

**Sample**	$M_{n\,(theory)}$ **(g mol⁻¹)**	$^{a}M_n$ **(g mol⁻¹)**	$^{b}M_w$ **(g mol⁻¹)**	$^{c}Đ$
$PFEMA_{100}$	17156	7,400	10,300	1.40
$PHMA_{33}FEMA_{67}$	17227	10,200	13,600	1.33
$PHMA_{50}FEMA_{50}$	17263	10,700	14,300	1.34
$PHMA_{60}FEMA_{40}$	17285	11,500	15,000	1.31
$PHMA_{67}FEMA_{33}$	17300	12,100	15,600	1.29
$PHMA_{80}FEMA_{20}$	17327	14,300	18,200	1.27
$PHMA_{90}FEMA_{10}$	17349	15,700	19,500	1.24
$PHMA_{100}$	17370	16,400	20,500	1.25

[a] Number-average molecular weight. [b] Weight-average molecular weight. [c] Dispersity, calculated as M_w/M_n.

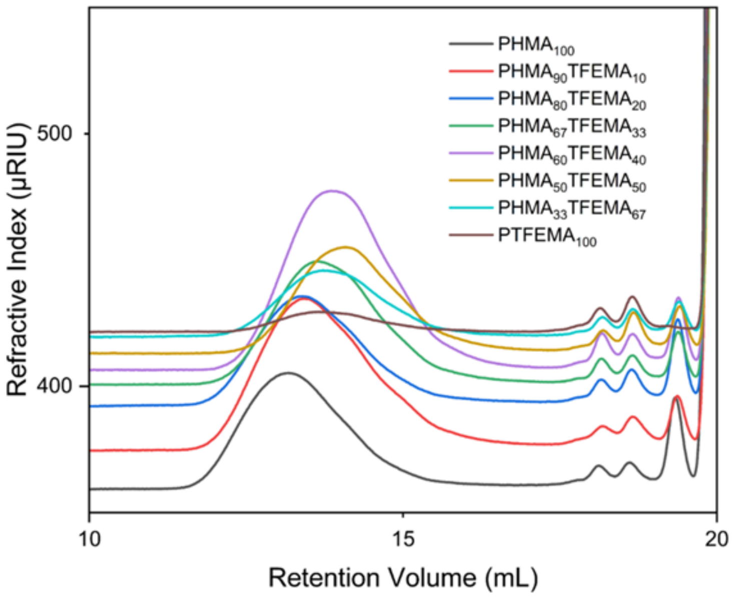

Figure S40. SEC traces for synthesised polymer series used to determine molecular weights

3.4 Thermal analysis

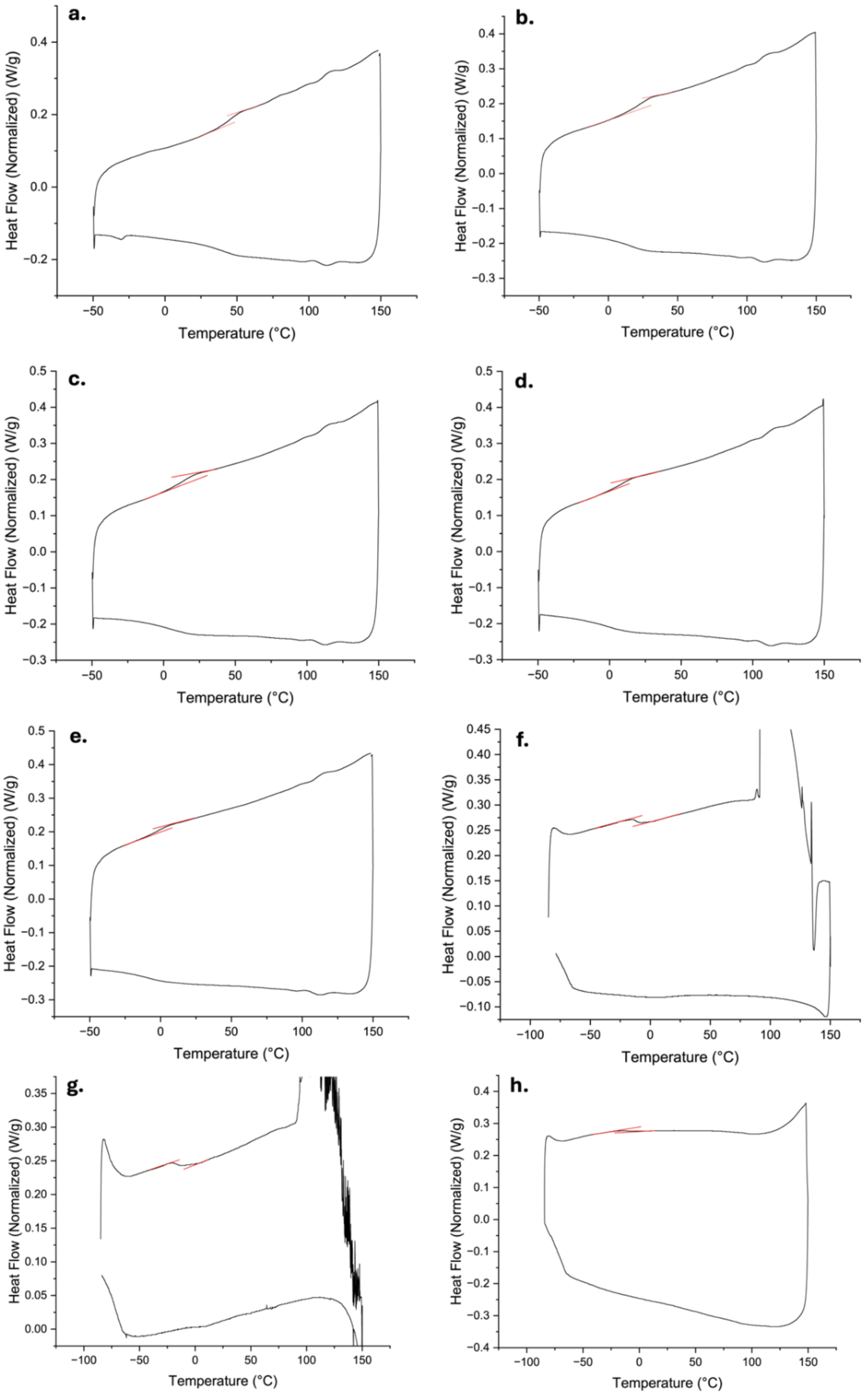

Figure S41. DSC traces for (a) $PTFEMA_{100}$, (b) $PHMA_{33}TFEMA_{67}$, (c) $PHMA_{50}TFEMA_{50}$, (d) $PHMA_{60}TFEMA_{40}$ (e) $PHMA_{67}TFEMA_{33}$, (f) $PHMA_{80}TFEMA_{20}$, (g) $PHMA_{90}TFEMA_{10}$ and (h)$PHMA_{100}$.

3.5 Steady-state optical properties

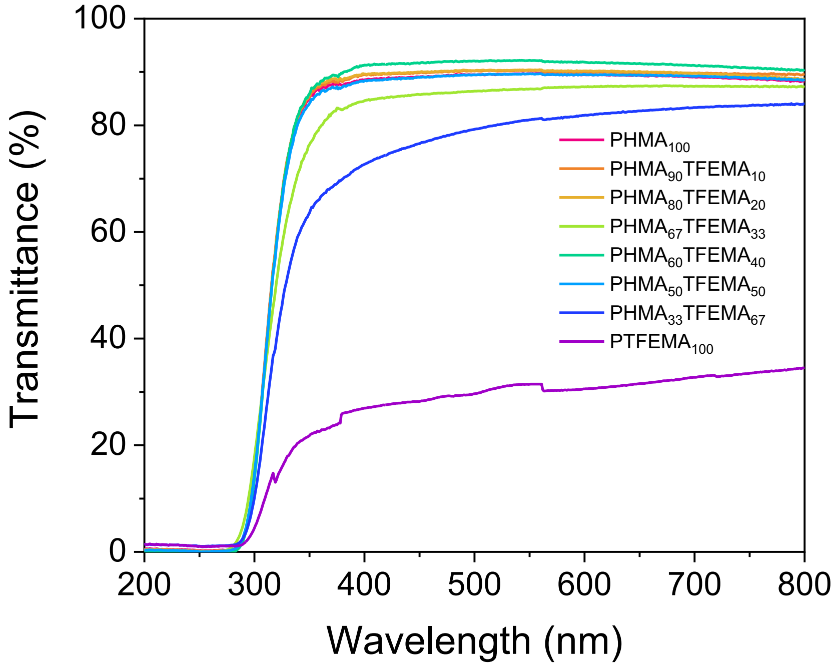

Figure S42. UV-visible transmittance spectra of end-reduced methacrylate (co)polymer films.

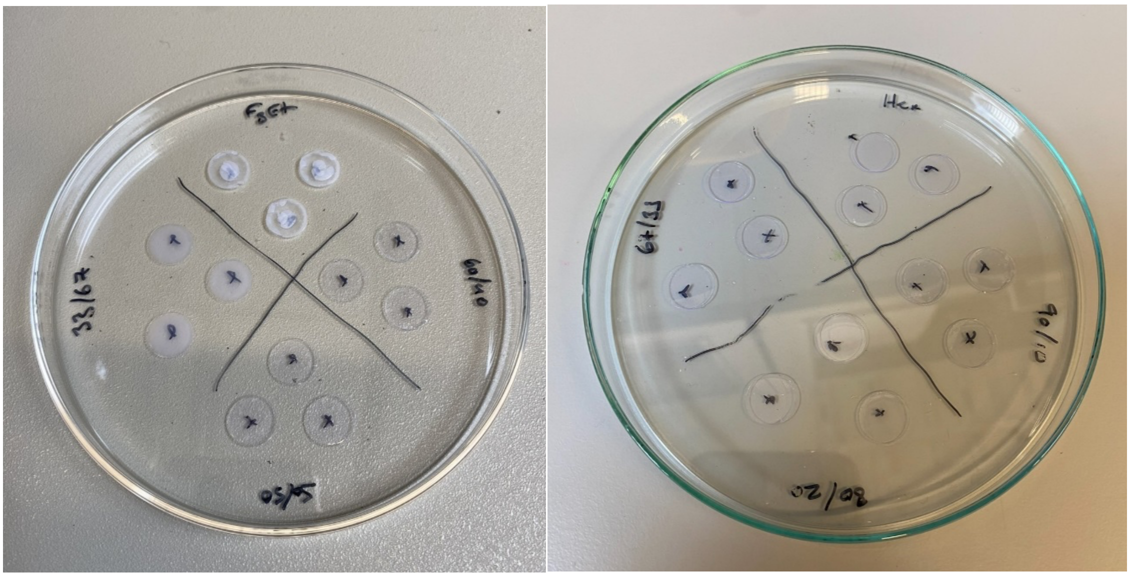

Figure S43. Photographs of undoped methacrylate (co)polymer films.

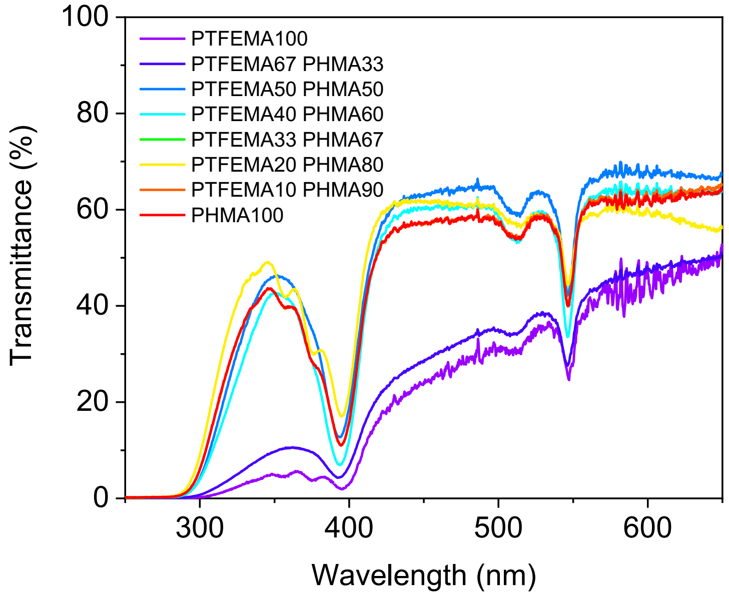

Figure S44. UV-visible transmittance of end-reduced methacrylate (co)polymer films doped with 30 mM DPA and 0.3 mM PdOEP.

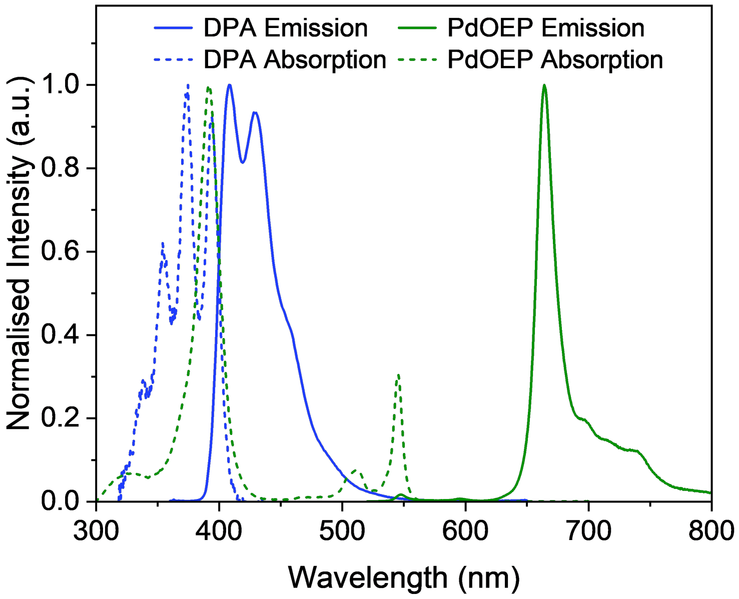

Figure S45. Normalised absorption (dashed lines) and emission spectra (solid lines) for the sensitiser PdOEP (green) and the emitter DPA (blue) in THF solution (5 μM). To measure the emission spectra, λ_{ex} was 350 nm for DPA and 505 nm for PdOEP. The PdOEP solution was deaerated by bubbling with N_2 for approximately 5 minutes prior to measurement of the emission spectrum.

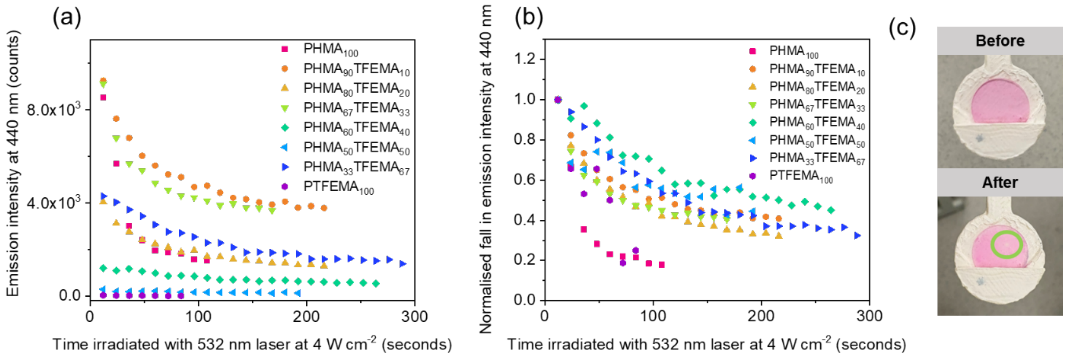

Figure S46. Photodegradation of methacrylate copolymer films under high power laser excitation (λ_{ex} = 532 nm at 4 W cm^{-2}). (a) Height of maximum upconverted emission intensity at 440 nm (peak of DPA emission) plotted against the time the sample has been irradiated. (b) Same data normalised to the highest value for each individual sample for easier comparison of the loss in emission over time between samples. (c) Photo of a representative sample PHMA$_{67}$TFEMA$_{33}$ after high power laser irradiation where a clear spot can be observed on the film where the laser hits. The effect is the same for all films tested.

Table S4. Photoluminescence quantum yield of DPA-only doped copolymer samples. The values were determined by integrating sphere method (λex = 375 nm).

**Polymer**	**Φ_{PL} (%)**
PHMA$_{100}$	*
PHMA$_{90}$TFEMA$_{10}$	89±1
PHMA$_{80}$TFEMA$_{20}$	92±3
PHMA$_{67}$TFEMA$_{33}$	96±2
PHMA$_{60}$TFEMA$_{40}$	88±1
PHMA$_{50}$TFEMA$_{50}$	89±1
PHMA$_{33}$TFEMA$_{67}$	92.5±0.6
PTFEMA$_{100}$	85±4

* sample form incompatible with sample holder used for measurements

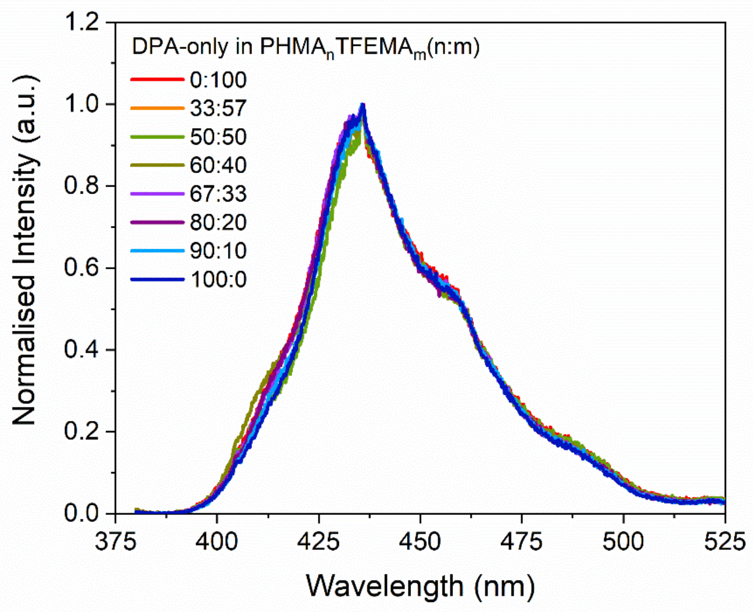

Figure S47. Normalised emission spectra of DPA-only (30 mM) doped in PHMA$_n$TFEMA$_m$ host matrices demonstrating DPA aggregation. Measurements were performed under ambient conditions (λex = 375 nm).

3.6 Fluorescence lifetimes

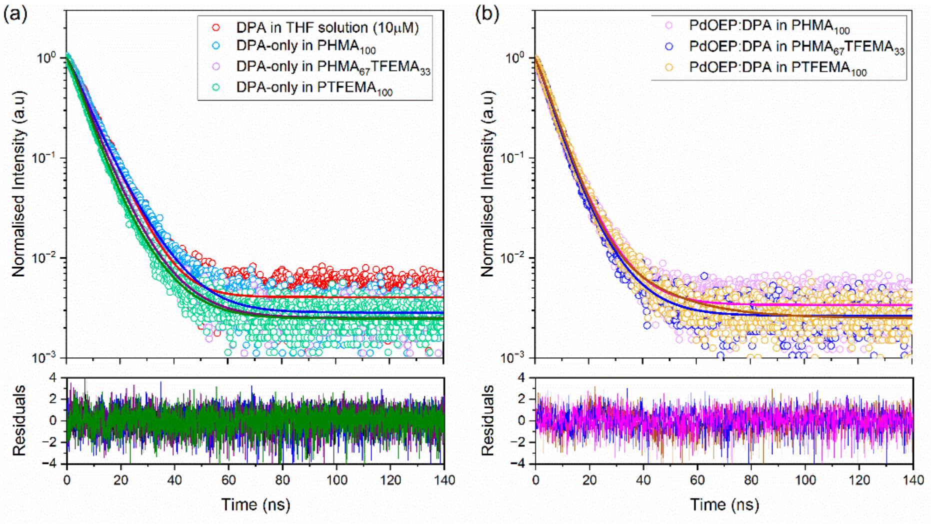

Figure S48: Fluorescence decay traces of (a) DPA in THF solution (at 10 μM concentration), DPA-only (30mM) doped in $PHMA_{100}$, $PHMA_{67}TFEMA_{33}$ and $PTFEMA_{100}$ host matrices and, (b) PdOEP:DPA (0.3mM:30mM) doped in $PHMA_{100}$, $PHMA_{67}TFEMA_{33}$ and $PTFEMA_{100}$ host matrices. Measurements were performed under ambient conditions, using a 375 nm excitation source and collecting at 440 nm.

Table S5: Fluorescence lifetimes of DPA in THF solution (10 μM concentration), DPA-only (30 mM) doped in $PHMA_{100}$, $PHMA_{67}TFEMA_{33}$ and $PTFEMA_{100}$ host matrices and PdOEP:DPA (0.3 mM:30 mM) doped in $PHMA_{100}$, $PHMA_{67}TFEMA_{33}$ and $PTFEMA_{100}$ host matrices. Measurements were performed under ambient conditions, using a 375 nm excitation source and detection at 440 nm.

**Sample**	**τ_1 (ns)/f_1(%)**	**τ_2 (ns)/ f_2(%)**	**<τ> (ns)**	**χ^2**
DPA in THF solution (10 μM)	7.47	-	-	1.138
DPA-only in $PHMA_{100}$	5.51/59	10.07/41	7.38	1.154
DPA-only in $PHMA_{67}TFEMA_{33}$	5.69/83	11.25/17	6.62	1.194
DPA-only in $PTFEMA_{100}$	5.02/78	10.25/22	6.15	1.107
PdOEP:DPA in $PHMA_{100}$	4.88/83	9.50/17	5.90	1.161
PdOEP:DPA in $PHMA_{67}TFEMA_{33}$	4.82/83	10.15/17	5.69	1.160
PdOEP:DPA in $PTFEMA_{100}$	5.45/94	18.04/6	6.18	1.250

3.7 Phosphorescence lifetimes

3.7.1 Ambient conditions

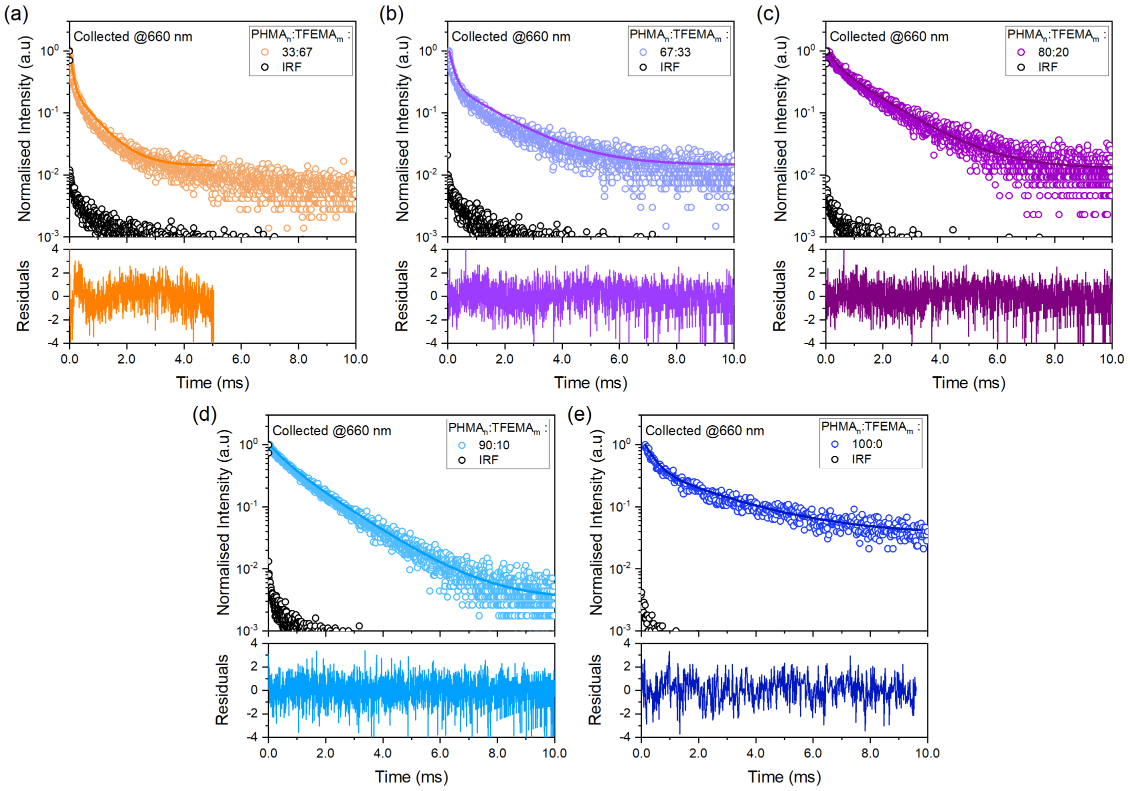

Figure S49. Phosphorescence decay traces of DPA:PdOEP (30 mM:0.3mM) doped in various polymer host matrices: (a) $PHMA_{33}TFEMA_{67}$, (b) $PHMA_{67}TFEMA_{33}$, (c) $PHMA_{80}TFEMA_{20}$, (d) $PHMA_{90}TFEMA_{10}$ and (e) $PHMA_{100}$. Measurements were performed in air using a 532 nm excitation source and collected at 660 nm.

Table S6. Phosphorescence lifetimes of PdOEP-only (0.3mM) doped in various polymer host matrices. Measurements were performed under ambient conditions, using a 532 nm excitation source and collecting at 660 nm.

**Polymer**	***Phosphorescence lifetimes (collection at 660 nm)***			
	**τ_1 (ms)/f_1 (%)**	**τ_2 (ms)/f_2 (%)**	**<τ> (ms)**	**χ^2**
$PHMA_{90}TFEMA_{10}$	0.33/28	0.61/72	0.53	1.160
$PHMA_{80}TFEMA_{20}$	0.32/42	0.63/58	0.50	1.166
$PHMA_{67}TFEMA_{33}$	0.36/5	0.92/95	0.89	1.186
$PHMA_{60}TFEMA_{40}$	0.12/61	0.54/39	0.28	1.227
$PHMA_{50}TFEMA_{50}$	0.08/41	0.67/59	0.43	1.195
$PHMA_{33}TFEMA_{67}$	0.20/62	0.51/38	0.32	1.146

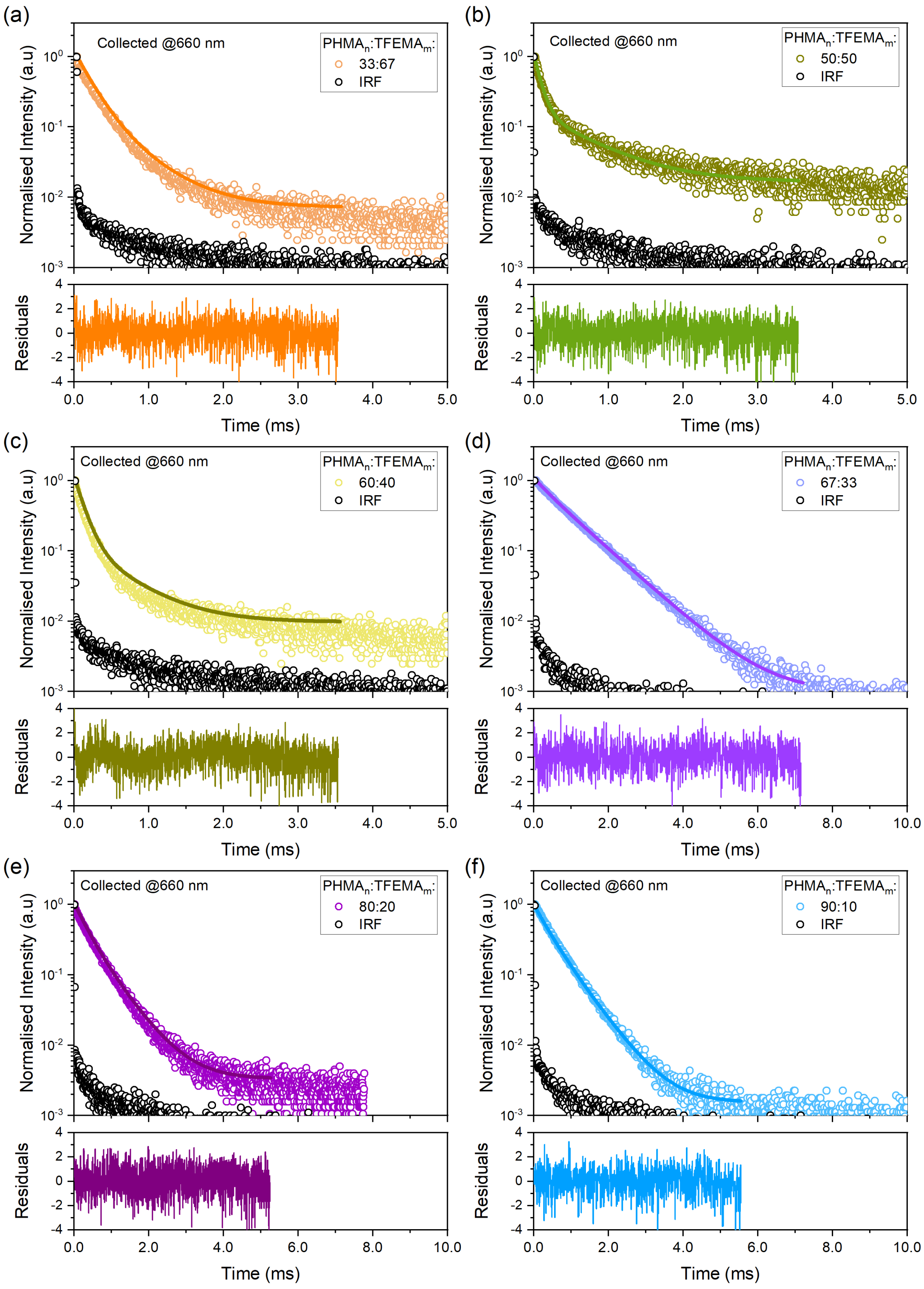

Figure 50. Phosphorescence decay traces of PdOEP-only (0.3mM) doped in various copolymer host matrices: (a) $PHMA_{33}TFEMA_{67}$, (b) $PHMA_{50}TFEMA_{50}$, (c) $PHMA_{60}TFEMA_{40}$, (d) $PHMA_{67}TFEMA_{33}$, (e) $PHMA_{80}TFEMA_{20}$, (f) $PHMA_{90}TFEMA_{10}$. Measurements were performed in air using a 532 nm excitation source and collected at 660 nm.

3.7.2 Nitrogen atmosphere

Table S7. Phosphorescence lifetimes of PdOEP-only (0.3mM) doped in both $PHMA_{100}$ and $PTFEMA_{100}$ host matrices, measured under nitrogen (N_2) purge for various durations, using a 532 nm excitation source and collecting at 660 nm.

Polymer	N_2 purging duration (minutes)	***Phosphorescence lifetimes (collection at 660 nm)***			
		τ_1 (ms)/f_1 (%)	τ_2 (ms)/f_2 (%)	<τ>(ms)	χ^2
$PHMA_{100}$	0	0.06/1	1.2/99	1.19	1.38
	30	0.15/3	1.2/97	1.17	1.463
	60	0.15/2	1.2/98	1.17	1.221
	120	0.31/3	1.2/97	1.17	1.203
$PTFEMA_{100}$	0	0.06/48	0.88/52	0.48	1.134
	30	0.24/11	1.60/89	1.45	1.163
	60	0.11/10	1.50/90	1.36	1.463
	120	0.10/10	1.50/90	1.36	1.191
	240	0.11/10	1.50/90	1.37	1.181

4 References

(1) Abel, B. A.; McCormick, C. L. Mechanistic Insights into Temperature-Dependent Trithiocarbonate Chain-End Degradation during the RAFT Polymerization of N -Arylmethacrylamides. *Macromolecules* **2016**, *49* (2), 465–474. https://doi.org/10.1021/acs.macromol.5b02463.

(2) Porrès, L.; Holland, A.; Pålsson, L. O.; Monkman, A. P.; Kemp, C.; Beeby, A. Absolute Measurements of Photoluminescence Quantum Yields of Solutions Using an Integrating Sphere. *Journal of fluorescence* **2006**, *16* (2), 267–273. https://doi.org/10.1007/S10895-005-0054-8.

(3) Ahn, T. S.; Al-Kaysi, R. O.; Müller, A. M.; Wentz, K. M.; Bardeen, C. J. Self-Absorption Correction for Solid-State Photoluminescence Quantum Yields Obtained from Integrating Sphere Measurements. *Review of Scientific Instruments* **2007**, *78* (8), 086105. https://doi.org/10.1063/1.2768926.

(4) De Mello, J. C.; Wittmann, H. F.; Friend, R. H. An Improved Experimental Determination of External Photoluminescence Quantum Efficiency. *Advanced Materials* **1997**, *9* (3), 230–232. https://doi.org/10.1002/adma.19970090308.

(5) Zhou, Y.; Castellano, F. N.; Schmidt, T. W.; Hanson, K. On the Quantum Yield of Photon Upconversion via Triplet–Triplet Annihilation. *ACS Energy Letters* **2020**, *5* (7), 2322–2326. https://doi.org/10.1021/acsenergylett.0c01150.

(6) Lakowicz, J. R. *Principles of Fluorescence Spectroscopy*; Springer, 2006. https://doi.org/10.1007/978-0-387-46312-4.
